# Standard length of peroral endoscopic myotomy (POEM) for achalasia: a systematic review and meta-analysis

**DOI:** 10.1093/dote/doae069

**Published:** 2024-08-30

**Authors:** Edoardo Vespa, Alberto Barchi, Francesco Vito Mandarino, Ernesto Fasulo, Maria Caterina Fratto, Sandro Passaretti, Francesco Azzolini, Edoardo Vincenzo Savarino, Silvio Danese

**Affiliations:** Department of Gastroenterology and Digestive Endoscopy, IRCCS Ospedale San Raffaele, Milan, Italy; Department of Gastroenterology and Digestive Endoscopy, IRCCS Ospedale San Raffaele, Milan, Italy; Department of Gastroenterology and Digestive Endoscopy, IRCCS Ospedale San Raffaele, Milan, Italy; Department of Gastroenterology and Digestive Endoscopy, IRCCS Ospedale San Raffaele, Milan, Italy; Department of Gastroenterology and Digestive Endoscopy, IRCCS Ospedale San Raffaele, Milan, Italy; Department of Gastroenterology and Digestive Endoscopy, IRCCS Ospedale San Raffaele, Milan, Italy; Department of Gastroenterology and Digestive Endoscopy, IRCCS Ospedale San Raffaele, Milan, Italy; Department of Surgery, Oncology, and Gastroenterology, University of Padua, Padua, Italy; Gastroenterology Unit, Department of Surgery, Oncology and Gastroenterology, Azienda Ospedale Università di Padova, Padua, Italy; Department of Gastroenterology and Digestive Endoscopy, IRCCS Ospedale San Raffaele, Milan, Italy; Faculty of Medicine, Department of Gastroenterology and Endoscopy, Università Vita-Salute San Raffaele, Milan, Italy

**Keywords:** achalasia, endoscopy, esophageal motility, myotomy

## Abstract

Peroral endoscopic myotomy (POEM) is an established treatment for achalasia, yet there is still a lack of technical standardization. No clear definition of ‘long’, ‘standard’, or ‘short’ POEM exists to date. We conducted a systematic review with meta-analysis to analyze current POEM length standards. We included studies reporting technical details of POEM, in which no definite or comparative myotomy length was intentionally adopted (standard myotomy). The primary outcome was the pooled mean total myotomy length. Sub-group analyses were performed to explore heterogeneity across studies. From the initial 7172 records, 31 studies with 3023 patients were included. Pooled mean of total myotomy length was 10.39 cm (95% CI 10.06–10.71; I^2^ 99.3%). Pooled mean of esophageal and gastric myotomy length, provided by 17 studies, was 7.11 cm (95% CI 6.51–7.71; I^2^ 99.8%) and 2.81 cm (95% CI 2.41–3-22; I^2^ 99.8%), respectively. On subgroup analysis for achalasia subtypes, pooled mean length in non-spastic achalasia (type I and II) was 10.17 cm (95% CI 9.91–10.43; I^2^ 94.2%), while in type III it was 14.02 cm (95% CI 10.59–17.44; I^2^ 98.9%). Pooled mean myotomy length for studies conducted between 2014–2020 was 10.53 cm (95% CI, 10.22–10.84; I^2^ 99.1%) and 9.74 cm (95% CI, 7.95–11.54; I^2^ 99.7%) in 2021–2022. Myotomy length during a ‘standard’ POEM is 10.4 cm, remaining over 10 cm in non-spastic achalasia. The high heterogeneity across studies confirms that the POEM technique needs further standardization. We found no significant time trend towards adopting short POEM, despite recent evidence supporting its use.

## INTRODUCTION

Achalasia is an esophageal motility disorder characterized by the absence of peristalsis in the esophageal body and poor relaxation of the lower esophageal sphincter (LES), mainly due to a degeneration in the inhibitory neurons located in the myenteric plexus.^1^ Typical symptoms are dysphagia, chest pain, and food regurgitation, with frequently associated weight loss as the disease progresses.^2^ Achalasia is a chronic, irreversible condition, and treatments are essentially palliative.^3^ Endoscopic management of achalasia has progressively developed over time: pneumatic dilation (PD) with air-filled balloon catheters represented the only efficient endoscopic treatment until the past decade.^4^ In 2010, Inoue and colleagues developed peroral endoscopic myotomy (POEM), which has soon revolutionized the landscape of achalasia treatment.^5^ POEM is an endoscopic procedure that involves the creation of a submucosal tunnel in the distal esophagus, through which electrosurgical dissection of the LES muscle fibers is performed. In spastic achalasia and non-achalasia hypercontractile disorders, myotomy can be liberally extended into the esophageal body, ablating spastic muscle segments. POEM is performed usually under general anesthesia, is technically more challenging, and requires higher costs compared to PD, in turn ensuring shorter recovery time and greater patient comfort over laparoscopic Heller myotomy (LHM), with high efficacy rates, both on long- and short-term, at the price of a likely higher gastroesophageal reflux disease (GERD) burden.^6^^,^^7^

While POEM has been increasingly used worldwide, there is still a lack of technical standardization, particularly regarding the extent of myotomy. The first POEM description implied an 8-cm myotomy (6 cm esophageal, 2 cm gastric).^5^ This length was not based on a functional outcome assessment, but more on a technical assurance that the LES would be entirely dissected. Being the LES the crucial site of obstruction, this arbitrary extension is in contrast with manometric findings describing its average length of only 3 cm.^8^ Barring type III achalasia, a short, LES-tailored POEM fulfills the physiological rationale of relieving the obstruction with efficacy, avoiding unnecessary submucosal tunneling and myotomy, and potentially reducing the risk of complications. Therefore, any myotomy longer than the actual LES extension (as long as it is confidently identified) may be unnecessary in type I and II achalasia. Moreover, there is evidence that myotomy limited to the LES (<6 cm) may be highly effective, timesaving, and associated with less post-operative GERD, yet it is common practice to use long myotomy in these patients.^9^^,^^10^

Since no clear definition of ‘long’, ‘standard’, or ‘short’ POEM exists to date, we conducted a systematic review and meta-analysis with the aim of clarifying current myotomy length standards during POEM procedures.

## METHODS

This systematic review and meta-analysis was designed according to the guidelines of the Preferred Reporting Items for Systematic Reviews and Meta-analyses (PRISMA) checklist (Supplementary Table 1)^11^ for meta-analysis, following an a priori established protocol. The study protocol was registered and accepted in the International Prospective Register of Systematic Reviews (PROSPERO),^12^ available at https://www.crd.york.ac.uk/prospero, (protocol code: CRD42023406828). No Institutional Review Board approval was needed.

### Search strategy and study selection

We performed a systematic literature search for published and studies on MEDLINE-Pubmed, Embase, Scopus, and Cochrane Library. The primary search strategy was designed using tools implemented in the above-mentioned databases. For Pubmed, MeSH terms were included in the search string. The following keywords were used as MeSH: ‘POEM’, ‘Myotomy’, and ‘Achalasia’. The detailed search string for all databases analyzed is shown in Supplementary Table 2. Only manuscripts in English and studies published in peer-reviewed journals from January 2008 until April 2023 (cut-off date) were included. The potential eligibility of articles was assessed by titles and abstracts. Then, full text of manuscripts was analyzed, and a final decision for inclusion was made after a detailed review of articles. Study selection was assessed by AB, and the evaluation of full texts of all relevant articles was performed by AB, EF, and MCF. Disagreements were resolved by consultation with a third investigator (EV).

### Eligibility criteria

We included observational studies and randomized controlled trials (RCTs) reporting on technical details of POEM for achalasia, specifically the myotomy length. Exclusion criteria were:

- age < 18 years
- not reporting the mean myotomy length or reporting mean myotomy lengths of only esophageal or gastric side
- diagnosing achalasia without high-resolution manometry
- including only patients with non-achalasia esophageal motility disorders
- comparing different myotomy lengths or with pre-specified myotomy lengths or with FLIP-tailored myotomy
- only including spastic (type III) achalasia
- case reports, case series, narrative, or systematic reviews and metanalyses
- not providing full-text, non-English language studies, or non-human studies.

For publications involving the same cohorts of patients, the most recent or largest cohort only was included. In absence of measures of statistical dispersion, particularly standard deviation (SD) or interquartile range (IQR), the study was not included.

### Data extraction

Three authors (AB, EF, and MCF) independently extracted data from eligible studies into a Microsoft Excel spreadsheet. Following data were extracted: author, publication year, country, mono-multicenter nature of the study with number of centers involved, study design, number of patients, age, sex, achalasia sub-type (I, II, and III according to Chicago classification^13–16^), previous achalasia treatment, previous botulin injection, previous PD, previous LHM, previous POEM, presence of sigmoid esophagus, anterior or posterior orientation of the myotomy, mean procedural time, mean post-procedural Eckardt score, mean follow-up time, mean total, esophageal and gastric myotomy length, clinical success rate, reflux symptoms rate, total rate of total adverse events (AEs), and serious AEs (according to ASGE Lexicon^17^).

### Outcomes

The primary outcome was the pooled total length of the myotomy (in centimeters, cm), including both the esophageal and gastric sides. Secondary outcomes were pooled rates of esophageal myotomy length, gastric myotomy length, clinical success (based on Eckardt score), total and serious (requiring further medical intervention during the same admission, admission to an intensive care unit, readmission, surgical conversion, or disability^17^) AEs, and the presence of reflux symptoms identifying a clinical suspicion of GERD.

Outcomes were stratified based on achalasia subtypes (per Chicago Classification version used in each study) to perform an a priori sub-group analysis.^13–16^ Further analyses were performed based on geographical localization, study design (RCT vs. observational, prospective vs. retrospective), and year of publication to explore heterogeneity. Particularly, concerning the study year, a sub-group analysis was performed stratifying studies based on the year (2021) of the first published RCT comparing short and long myotomy by Nabi and colleagues,^9^ showing comparable efficacy of short POEM, to assess if a trend towards short myotomy adoption was present.

### Risk of bias

Two review authors (EV and AB) assessed the risk of bias by using the Newcastle–Ottawa scale (NOS) for assessing the quality of non-randomized studies in meta-analysis.^18^ According to the score, all studies were evaluated by three perspectives: the selection of the study groups, the comparability of groups, and the ascertainment of the outcome. NOS results were converted into the Agency for Healthcare Research and Quality (AHRQ) standards of ‘good’, ‘fair’, and ‘poor’ quality. In particular, since conversion guidelines exist, conversion thresholds were considered, from prior publication,^19^ as follows: ‘Good quality’: 3 or 4 points in selection domain and 1 or 2 points in comparability domain and 2 or 3 points in outcome/exposure domain; ‘Fair quality’: 2 points in selection domain and 1 or 2 points in comparability domain and 2 or 3 points in outcome/exposure domain; ‘Poor quality’: 0 or 1 point in selection domain or 0 points in comparability domain or 0 or 1 point in outcome/exposure domain. In case of disagreements, resolution was resolved by discussion with a third reviewer (FVM).

### Statistical analysis

All analyses were performed using statistical software Open Meta-analyst (CEBM; Brown University, Rhode Island, USA), including generation of forest plots and calculation of confidence intervals (CIs). Categorical data were expressed as proportions. Proportions were expressed as the total number of events on the total number of patients and computed as raw proportions or natural logarithm proportions. Continuous variables were expressed as mean plus standard deviation (SD). Studies providing median plus range with reference to maximum and minimum of the whole distribution, were still included prior estimation of mean and SD following Hozo formula.^20^ Studies managing non-normal distributed data and providing median and interquartile range (IQR), were included in the pooled analysis prior to estimation of mean and SD using the Shi and Luo method^21^^,^^22^: first, the skewness from normality was assessed as suggested by Shi *et al.*^23^ If the distribution resulted in not significantly skewed from normality, mean and SD were estimated as suggested by Luo *et al*.^22^ and Wan *et al.*,^24^ respectively. If the distribution resulted significantly skewed from normality, the study was excluded from the pooled analysis. The meta-analysis was conducted without taking into account data homogeneity, setting a CI of 95% for all measures. The homogeneity of effect sizes among pooled studies was assessed with the I^2^ statistic and Chi-Square test (χ^2^). According to Higgins *et al*.,^25^ I^2^ was interpreted as follows: insignificant heterogeneity if for I^2^ 0%–25%, low heterogeneity for I^2^ 25%–50%, moderate heterogeneity for I^2^ 50%–75% and high heterogeneity when I^2^ was >75%.^25^ Fixed or random effects were applied according to values of heterogeneity in each analysis (<50% or >50% values, respectively). The random effects model described by DerSimonian and Laird was used for all analyses. Sensitivity analyses by a priori subgroup analyses for different variables were performed to explore data heterogeneity source, and leave-one-out analyses were explored to identify each study weight on each effect measure.

## RESULTS

### Characteristics of studies and included patients

**Fig. 1:**
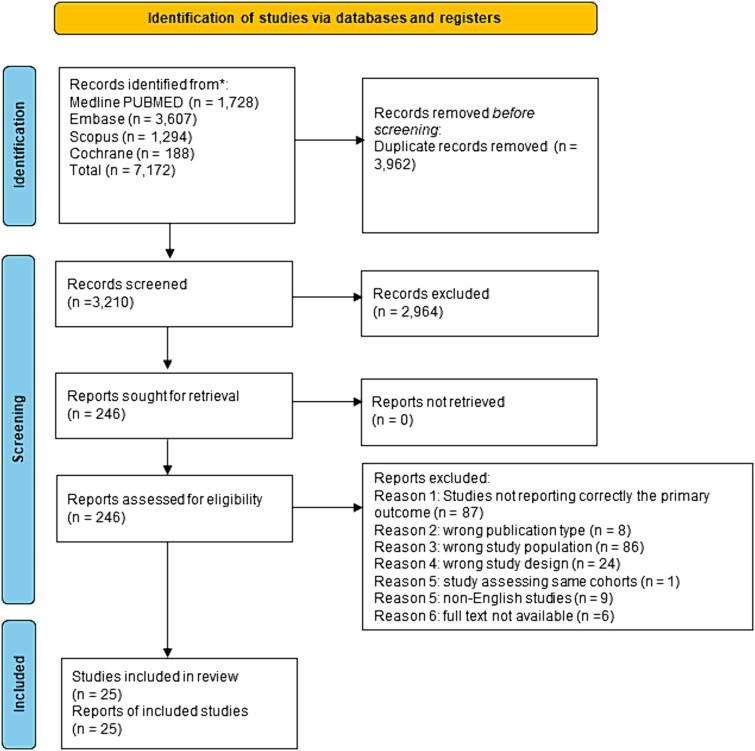
Flow diagram of study selection according to the preferred reporting items for systematic reviews and meta-analyses.

From the initial 7172 records, upon applying exclusion criteria, a total of 25 full articles were included. Of those, six provided results on the primary outcome in separate subgroups and were therefore included as separate studies. Overall, data from 31 cohorts were analyzed.^7^^,^^26–49^ The complete flowchart of the systematic review according to PRISMA guidelines is depicted in Fig. 1. Of the 25 full papers included, 11 studies were performed in Eastern countries (1848 patients); 11 studies were based in Western countries (804 patients). Four studies were multicentric, while the remainder were monocentric. Seventeen had a retrospective design, two were RCTs, and six were prospective studies. Study characteristics are summarized in Table 1.

**Table 1:** Baseline characteristics of the included studies

**Author**	**Year**	**Country**	**Study design**	**Centers involved**	**Pts (n)**	**Male, n (%)**	**Age,** **mean (SD)**	**Achalasia subtype, n**	**Previous treatment, n (%)**	Type of treatment, n*	**Mean baseline IRP (SD)**	**Mean baseline Eckardt (SD or range, IQR)**	**Ref**
Chang et al.	2019	Korea, Turkey	Retrospective	Multicenter	195	93 (47.6)	43.7 (15.53)	I: 83, II: 91, III: 21	85 (43.5)	Bot: 21 PD: 36 HM:4 POEM:5	11.7 (7.6)	6.3 (2.3)	26
Chen et al.	2014	China	Retrospective	Single center	45	16 (35.5)	46.3 (19.1)	I: 14, II: 24, III: 7	NA	NA	NA	NA	27
Arshava et al.	2018	USA	Retrospective	Single center	31	19 (61.2)	47.5 (NA)	I: 7, II: 18, III: 6	18 (58.1)	Bot: 5 PD: 9 HM:4 POEM:0	NA	Median 6,3 [3–10]	29
De Pascale et al.	2017	Italy	Retrospective	Multicenter	32	20 (62.5)	Median 56 [18–83]	I: 0, II: 31, III: 1	5 (15.6)	Bot: 2 PD: 3 HM:0 POEM:0	NA	Median 6 [5–12]	28
DeWitt et al.	2022	USA	Retrospective	Single center	87	50 (57.4)	51.3 (17)	I: 10, II: 68, III: 9	0 (0)	Bot: 0 PD: 0 HM:0 POEM:0	32.5 (12.2)	8.1 (1.7)	30
Qiu et al.	2021	China	Retrospective	Single center	112	62 (55.3)	44.8 (13.4)	I: 47, II: 63, III: 2	34 (30.3)	Bot: 7 PD: 20 HM:4 POEM:4	NA	8.0 (NA)	31
Guo et al.	2017	China	Retrospective	Single center	67	36 (53.7)	40.7 (15.3)	I: 13, II: 50, III: 4	17 (25.4)	Bot: 2 PD: 12 HM:3 POEM:0	27.6 (8.1	7.6 (2.3)	32
Karyampudi et al.	2020	India	Retrospective	Single center	50	31 (62)	Median 41.5 [36.5–52]	I: 8, II: 38, III: 4	10 (20)	Bot: 0 PD: 10 HM:0 POEM:0	Median 35.7 [24.2–38.7]	median 7 [6–8]	33
Liu et al. 1	2019	China	Retrospective	Single center	139	65 (46.7)	70.2 (5.7)	I: NA, II: NA, III: NA	34 (24.4)	Bot: 21 PD: 36 HM:4 POEM:5	NA	7.2 (1.9)	34
Liu et al. 2	2019	China	Retrospective	Single center	275	127 (46.1)	42.1 (12.8)	I: NA, II: NA, III: NA	75 (27.3)	Bot: 21 PD: 36 HM:4 POEM:5	NA	7,1 (1.9)	34
Khashab et al. 1	2020	USA, Greece, Japan, China	RCT	Multicenter	71	37 (52.1)	Median 54 [32–66]	I: 13, II: 48, III: 10	25 (35.2)	Bot: 7 PD: 18 HM:0 POEM:0	28 [20–36]	Median 8 [6–9]	35
Khashab et al. 2	2020	USA, Greece, Japan, China	RCT	Multicenter	77	43 (55.8)	Median 51 [36–62]	I: 5, II: 58, III: 14	25 (32.5)	Bot: 5 PD: 20 HM:0 POEM:0	27 [18–35]	Median 8 [6–9]	35
Yang et al.	2015	USA	Prospective	Single center	52	25 (48.1)	Median 55 [20–83]	I: 9, II: 34, III: 9	23 (44.2)	Bot: 10 PD: 6 HM:1 POEM:0	NA	Median 8 [3–14]	36
Sanaka et al. 1	2020	USA	Retrospective	Single center	93	93 (100)	Median 52 [43–58]	I: 29, II: 45, III: 10	30 (32.2)	Bot: 12 PD: 49 HM:26 POEM:0	22.9 [13.1–34.2]	Median 7 [5–8]	37
Sanaka et al. 2	2020	USA	Retrospective	Single center	55	55 (100)	Median 74 [70–79]	I: 13, II: 24, III: 11	17 (31.0)	Bot: 21 PD: 21 HM:4 POEM:0	22.5 [15.7–30.5]	Median 6 [4–8]	37
Raja et al.	2018	USA	Prospective	Single center	152	81 (53.2)	Median 57.8 [48.8–73.2]	I: 41, II:69, III: 25	0 (0)	Bot: 0 PD: 0 HM:0 POEM:0	NA	Median 7 [5–9]	38
Nast et al.	2018	Germany	Retrospective	Single center	114	69 (60.5)	47.3 (16.9)	I: 28, II: 72, III: 18	53 (46.5)	Bot: 17 PD: 65 HM:1 POEM:0	NA	NA	39
Hu et al.	2014	China	Prospective	Single center	32	17 (53.1)	Median 43.6 [18–72]	I: NA, II: NA, III: NA	23 (71.8)	Bot: 3 PD: 14 HM:3 POEM:0	NA	Median 7.8 [4–12]	40
Dacha et al.	2018	USA	Retrospective	Single center	62	26 (41.9)	Median 59 [36–85]	I: 13, II: 47, III: 1	32 (51.6)	Bot: 0 PD: 0 HM:0 POEM:0	NA	9.3 (1.5)	42
Teh et al.	2021	Singapore	Prospective	Single center	58	30 (51.7)	51.4 (14.3)	I: 16, II: 34, III: 3	20 (34.5)	Bot: 4 PD: 19 HM:5 POEM:0	23.5 (33.1)	6.1 (2.4)	41
Evensen et al.	2021	Norway	Prospective	Single center	50	24 (48)	Median 47 [24–70]	I: 7, II: 39, III: 4	NA	NA	Median 33.9 [26–38.8]	Median 8 [6.8–9.3]	43
Ichkhanian et al. 1	2020	USA, France, Greece, Hong Kong, Japan	Prospective	Multicenter	54	25 (46.2)	52.3 (21)	I: 13, II: 33, III: 8	NA	NA	NA	Median 8 [6–9]	44
Ichkhanian et al. 2	2020	USA, France, Greece, Hong Kong, Japan	Prospective	Multicenter	57	34 (59.6)	51.2 (18)	I: 4, II: 42, III: 11	NA	NA	NA	Median 8 [7–9]	44
Mondragòn et al.	2019	Mexico	Prospective	Single center	45	16 (35.5)	Median 45.5 [22–69]	I: 12, II: 24, III: 9	9 (20)	Bot: 3 PD: 1 HM:5 POEM:0	Median 25.3 [17–55]	Median 10 [8–12]	45
Xu et al. 1	2020	China	Retrospective	Single center	38	38 (100)	41.9 (13.2)	I: 2, II: 36, III: 0	NA	NA	28.1 (10.7)	Median 5 [3.7–5.2]	46
Xu et al. 2	2020	China	Retrospective	Single center	40	0 (0)	45.3 (12.2)	I: 7, II: 32, III: 1	NA	NA	33 (11.8)	Median 5 [4–6]	46
Wang et al. 1	2022	China	Retrospective	Single center	579	249 (43.0)	Median 45.7 [18–85]	I: 113, II: 428, III: 38	112 (19.3)	Bot: 37 PD: 61 HM:9 POEM:5	NA	Median 7 [4–12]	47
Wang et al. 2	2022	China	Retrospective	Single center	123	60 (48.7)	Median 43.3 [19–77]	I: 26, II: 90, III: 7	34 (27.6)	Bot: 10 PD: 20 HM:1 POEM:1	NA	Median 7,2 [4–12]	47
Farias et al.	2019	Brazil	Retrospective	Single center	31	15 (48.3)	44.6 (14.8)	I: NA, II: NA, III: NA	5 (16.1)	Bot: 0 PD: 2 HM:3 POEM:0	NA	Median 8 [6–9]	48
Tang et al.	2017	China	Retrospective	Single center	95	46 (48.4)	37.7 (11.6)	I: 29, II: 62, III: 4	30 (31.6)	Bot: 4 PD: 26 HM:0 POEM:0	NA	5.7 (2.1)	49
Werner et al.	2019	Germany	RCT	Multicentric	112	68 (60.7)	48.6 (14.9)	I: 15, II: 85, III: 12	39 (34.9)	Bot: 12 PD: 32 HM:0 POEM:0	NA	6.8 (2)	7

SD, standard deviation; RCT, randomized controlled trial; IRP, integral relaxation pressure; IQR, interquartile range.

The 31 different cohorts’ studies reported outcomes of 3023 patients treated with POEM for esophageal achalasia. Median follow-up time was 45 months (IQR 24–65). In the whole population, 51.9% were men, and the mean age was 49.4 ± 8.0 years. The presence of sigmoid esophagus was reported in 12 studies (146/1139, 12.8%). Manometric sub-type according to Chicago classification^13–15^^,^^50^ was reported in 27 studies (2495 total patients). The most frequent manometric subtype reported was type II (1685 patients, 67.5%), followed by type I (577 patients, 23.1%), while 249 patients (9.9%) had type III achalasia. Four studies (477 patients, 19.1%) did not specify the achalasia subtype. Of 2739 patients (25 studies), 755 had received at least one prior treatment (27.5%): LHM in 77 (2.8%), PD in 449 (16.3%), prior POEM in 15 (0.5%). The mean procedural time (reported by 24 studies) was 73.6 ± 29.7 minutes. In 12 studies (674 patients), orientation of myotomy was specified. Anterior approach was used in 281 patients (41.6%), 384 posterior (56.9%). Procedural patient characteristics of each study are provided in Table 2.

**Table 2:** Procedural characteristics

**Author**	**Year**	**Pts (n)**	**Sigmoid esophagus, n (%)**	**Myotomy orientation, n**		**Total myotomy length, cm (SD)**	**Esophageal myotomy length, cm (SD)**	**Gastric myotomy length, cm (SD)**	**Procedural time, min (SD or range, IQR)**	**Mean follow-up time, days (SD or range, IQR)**
				**Anterior**	**Posterior**					
Chang, et al.	2019	195	17 (8.7)	NA		8.9 (2.2)	NA	NA	NA	288.8 (255.3)
Chen, et al.	2014	45	NA	NA		9.6 (1.5)	NA	NA	NA	NA
Arshava, et al.	2018	31	NA	NA		12.7 (1.4)	NA	NA	NA	288 (NA)
De Pascale, et al.	2017	32	NA	NA		11.7 (0.8)	8.9 (1.2)	3 (0.5)	Median 63.4 [32–114]	792 (256.5)
DeWitt, et al.	2022	87	NA	14 (16.1)	64 (73.5)	9.7 (2.9)	NA	NA	NA	216 (66)
Qiu, et al.	2021	112	NA	NA		11.2 (0.5)	NA	NA	Median 45.5 [35.8–60.3]	975 (129)
Guo, et al.	2017	67	NA	NA		10.7 (2.3)	NA	NA	43.2 (35.7)	40.1 (2.8)
Karyampudi, et al.	2020	50	NA	10 (20)	40 (80)	11.7 (3.7)	7.7 (3.1)	2.8 (0.5)	Median 38 [30.7–46.2]	NA
Liu, et al. 1	2019	139	16 (11.5)	NA		10.6 (1.8)	8.5 (1.8)	2.1 (0.4)	Median 50 [36–76]	1260 (68)
Liu, et al. 2	2019	275	33 (12)	NA		10.2 (1.8)	8.1 (1.2)	2.1 (0.3)	Median 50 [36–70]	1260 (68)
Khashab, et al. 1	2020	71	6 (13.3)	71 (100)	0 (0)	10.1 (0.2)	7.1 (0.2)	2.9 (0.2)	Median 59 [46–100]	NA
Khashab, et al. 2	2020	77	9 (16.0)	0 (0)	77 (100)	10 (0.2)	7.1 (0.2)	2.9 (0.2)	Median 67 [43–109]	NA
Yang, et al.	2015	52	NA	NA		13.1 (2.4)	10.2 (1.8)	3.1 (0.4)	Median 90.6 [32–214]	30 (NA)
Sanaka, et al. 1	2020	93	NA	NA		9 (0.3)	5 (0.4)	3.9 (0.2)	Median 98 [74–114]	68 (78.3)
Sanaka, et al. 2	2020	55	NA	NA		9.5 (0.5)	5.1 (0.2)	4 (0.1)	Median 90 [67.5–110]	Median 71 [67–82]
Raja, et al.	2018	152	0 (0)	NA		9 (0.3)	5 (0.4)	3.9 (0.2)	Median 96 [77–114]	NA
Nast, et al.	2018	114	NA	NA		10.9 (3.6)	NA	NA	Median 106.8 [32.7]	NA
Hu, et al.	2014	32	32 (100)	0 (0)	32 (100)	10.3 (1.7)	8 (1.4)	2.6 (0.7)	Median 63.7 [22–130]	900 (150)
Dacha, et al.	2018	62	5 (8)	0 (0)	62 (100)	7.5 (0.8)	NA	NA	77,6 (27.3)	365 (NA)
Teh, et al.	2021	58	14 (24.1)	46 (79.3)	12 (20.7)	11.8 (2.4)	NA	NA	137,3 (54.4)	1095 (NA)
Evensen, et al.	2021	50	9 (18.3)	50 (100)	0 (0)	11.5 (0.6)	NA	NA	Median 153 [130–185]	365 (NA)
Ichkhanian, et al. 1	2020	54	NA	54 (100)	0 (0)	10.8 (2.3)	7.4 (2.8)	3 (1.3)	Median 63 [49–101]	730 (207)
Ichkhanian, et al. 2	2020	57	NA	0 (0)	57 (100)	10.4 (2.3)	7.1 (2.4)	2.8 (2.1)	Median 64 [46–107]	730 (156)
Mondragòn, et al.	2019	45	5 (11.1)	36 (80)	9 (20)	13 (3.5)	NA	NA	Median 54 [37–77]	365 (NA)
Xu, et al. 1	2020	38	NA	NA		10.9 (2)	NA	NA	NA	NA
Xu, et al. 2	2020	40	NA	NA		11 (2.5)	NA	NA	NA	NA
Wang, et al. 1	2022	579	NA	NA		7.1 (1.9)	5.1 (2.1)	2 (0.7)	Median 43.5 [17–180]	715.3 (427.5)
Wang, et al. 2	2022	123	NA	NA		7.1 (4.1)	5.3 (4.1)	1.9 (0.6)	Median 54.6 [22–170]	773.1 (472.5)
Farias, et al.	2019	31	0 (0)	0 (0)	31 (100)	11.3 (1.2)	7.7 (1.2)	3.7 (0.7)	99.2 (33.3)	365
Tang, et al.	2017	95	NA	NA		9.9 (3.6)	6.9 (3)	2.9 (1.1)	59.8 (24.2)	767.1 (NA)
Werner, et al.	2019	112	NA	NA		12.5 (3)	NA	NA	NA	NA

SD, standard deviation; IQR, interquartile range; NA, not available.

### Quality and publication bias assessment outcomes

Concerning 25 included full papers, the mean NOS score was 4.5 (range 3–7). Overall, 13 studies resulted in poor quality,^27^^,^^29^^,^^30^^,^^33^^,^^36–39^^,^^43^^,^^45^^,^^46^^,^^48^^,^^49^ 6 studies of fair quality,^26^^,^^28^^,^^31^^,^^34^^,^^41^^,^^47^ and the remaining 6 studies of good quality.^7^^,^^32^^,^^35^^,^^40^^,^^42^^,^^44^ Only three studies reported of good quality were retrospective analyses, while one study was a multicenter prospective clinical study, one study was monocentric prospective study, and two were RCTs, the only studies scoring one point for reporting comparability of cohorts on the basis of the study design. The detailed NOS/AHRQ score is reported in Supplementary Table 3. Publication bias was assessed for every study cohort analyzed. At the publication bias assessment for the primary outcome of mean myotomy length, no publication bias was detected between the included studies, with an Egger test score of 1.2 (*P* = 0.2). A funnel plot of publication bias assessment for the primary outcome is shown in Supplementary Fig. 1.

### Study outcomes concerning myotomy characteristics

**Fig. 2:**
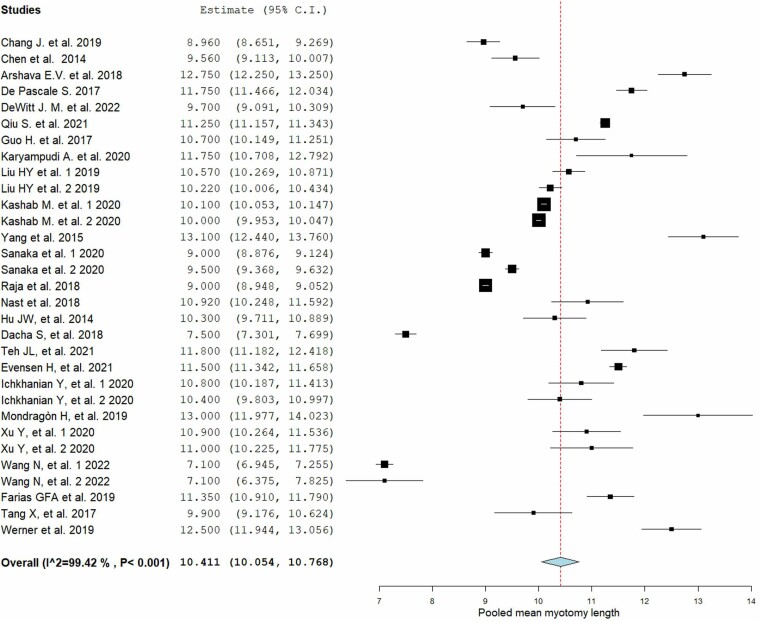
Pooled mean total myotomy length.

The pooled mean total length of myotomy was 10.41 cm (95% CI 10.05–10.76; I^2^ 99.4%), reported by all studies (Fig. 2). The pooled means of esophageal and gastric myotomy lengths, provided by 17 studies, were 7.05 cm (95% CI 6.46–7.64; I^2^ 99.8%) and 2.91 cm (95% CI 2.50–3.33; I^2^ 99.8%), respectively (Supplementary Fig. 2). The heterogeneity was not found to arise from a single study after performing leave-one-out analysis for both primary and secondary outcomes (Supplementary Fig. 3). At the subgroup analysis for achalasia subtypes, pooled mean length in non-spastic achalasia type I was 10.11 cm (95% CI 9.68–10.53; I^2^ 92.7%), in type II attested at 10.59 cm (95% CI 10.33–10.85; I^2^ 96.4%), while in spastic type III resulted in 14.12 cm (95% CI 10.97–17.27; I^2^ 99.8%) (Supplementary Fig. 4). The myotomy lengths in different achalasia subtypes are summarized in Supplementary Table 4. On sub-group analysis based on study design, the mean pooled total myotomy length in RCTs was 10.43 cm (95% CI, 10.15–10.70; I^2^ 97.1%) and in observational studies was 10.38 cm (95% CI, 9.85–10.91; I^2^ 99.7%). Based on design, in retrospective and prospective observational studies, it was 10.06 cm (95% CI 9.34–10.78; I^2^ 99.4%) and 11.21 cm (95% CI, 9.98–12.44; I^2^ 99.4%), respectively.

At a median follow-up of 29 months, in 18 studies, the pooled clinical success rate was 91% (95% CI 88.9%–93.2%; I^2^ 67.2%) (Supplementary Fig. 5), while the pooled rate of post-POEM clinical reflux was 26.2% (95% CI 21.0%–31.3%; I^2^ 89.5%) (Supplementary Fig. 6). Based on study design, RCTs reported a pooled clinical success rate of 88.3% (95% CI, 84.5–92.4; I^2^ 0%), while retrospective and prospective cohort studies both showed a pooled clinical success rate of 91.5% (95% CI, 89.1–93.9; I^2^ 58.5%) and 91.5% (95% CI, 86.6–96.6; I^2^ 71.3%). The pooled rate of reflux symptoms for retrospective, prospective, and RCTs was 19.5% (95% CI, 13.9–27.5; I^2^ 92.1%), 30.6% (95% CI, 22.5–41.5; I^2^ 75.7%), and 42.8% (95% CI, 36.8–49.8; I^2^ 0%), respectively. AEs were reported in 19 studies, with an overall pooled rate of 16% (95% CI 12%–19%; I^2^ 91.5%). Severe AEs pooled rate was 1.2% (95% CI 0.6%–1.9%; I^2^ 14.2%) (Supplementary Fig. 7). Retrospective, prospective, and RCTs showed pooled AEs rates of 11.8% (95% CI, 7.5–18.3; I2 91.8%), 30.9% (95% CI, 21.0–45.4; I2 82.4%), and 10.4% (95% CI, 7.3–14.9; I^2^ 0%). Descriptive post-procedural outcomes are summarized in Supplementary Table 5.

### Geographic trends

**Fig. 3:**
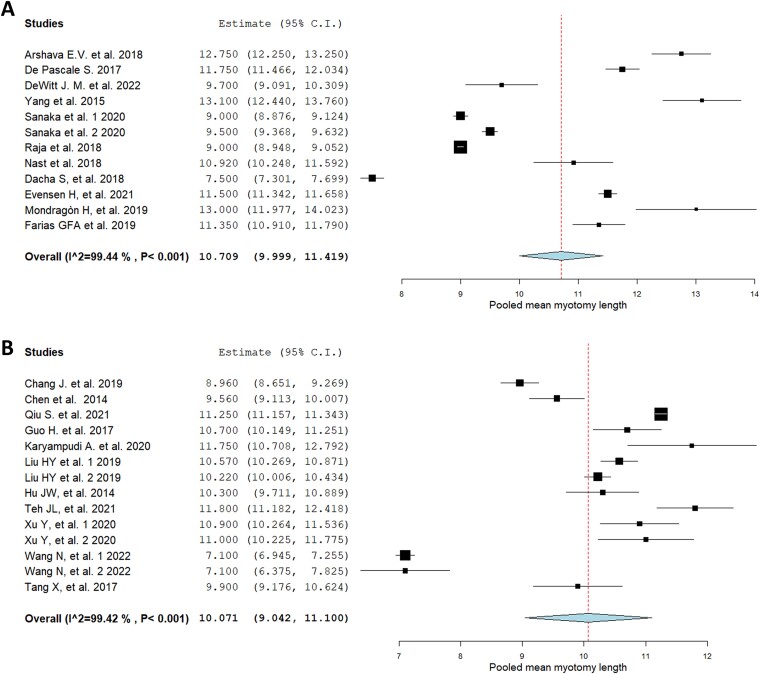
Geographical sub-group analysis of myotomy lengths: western countries (A) and eastern countries (B).

No geographical difference was observed: in Western countries (11 studies) mean myotomy length was 10.70 cm (95% CI 9.99–11.41; I^2^ 99.4%) (Fig. 3A), while in Eastern countries (11 studies) mean myotomy length was 10.07 cm (95% CI 9.04–11.01; I^2^ 99.4%) (Fig. 3B). Data on total myotomy length were available from nine different countries, either eastern or western. China was the most represented country with eight studies; the United States had 6. Details of myotomy lengths in different countries are summarized in Supplementary Table 6.

### Temporal trends

**Fig. 4:**
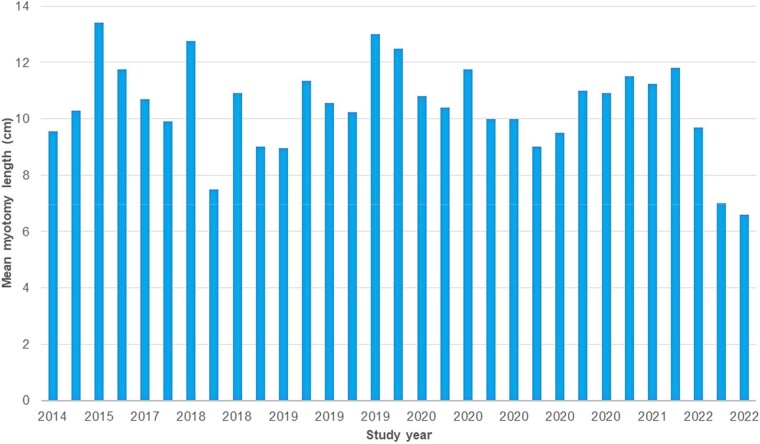
Timeline for trend in myotomy lengths in POEM studies from inception to 2022.

Looking at the temporal trend regarding the primary outcome, pooled myotomy lengths were analyzed per year of publication and divided into subgroups of year spans. The first article included in the meta-analysis dated to 2014, while the latest to 2022; therefore, an 8-year time span was considered. At a per-year analysis, in 2014 (two studies) reported a pooled mean total myotomy length of 9.90 cm (95% CI 9.18–10.62; I^2^ 74.02%), in 2015 (1 study) it was 13.10 cm (95% CI 12.44–13.76), in 2017 (3 studies) 10.82 cm (95% CI 9.73–11.91; I^2^ 92.95%), in 2018 (4 studies) 10.01 cm (95% CI 8.59–11.52; I^2^ 99.4%), in 2019 (6 studies) 11.03 (95% CI 10.12–11.93; I^2^ 97.3%), in 2020 (9 studies) 10.14 cm (95% CI 9.85–10.42; I^2^ 97.7%), in 2021 (3 studies) 11.41 cm (95% CI 11.17–11.66; I^2^ 78.8%), while in 2022 (2 studies) it was 7.95 cm (95% CI 6.34–9.57; I^2^ 96.9%). Pooling studies published before and after 2021, the year of the first trial of Nabi and colleagues on short versus long myotomy comparison, the mean total myotomy length was 10.53 cm (95% CI, 10.22–10.84; I^2^ 99.1%) until 2020, while it was 9.74 cm (95% CI, 7.95–11.54; I^2^ 99.7%) in 2021–2022 (Supplementary Fig. 8). The complete temporal trend of total myotomy length in POEM is shown in Fig. 4.

## DISCUSSION

Our systematic review and meta-analysis focused on current myotomy length standards during POEM. As we only included studies performing an ‘uncontrolled’ POEM in terms of length, we propose that the pooled mean total myotomy length of 10.4 cm, demonstrated in our study, should be considered the ‘standard’ POEM length in clinical practice. Another relevant finding is the high heterogeneity (I^2^: 99.3%) of the total myotomy length across studies, which emphasizes a lack of consensus, thereby confirming that POEM technique requires further standardization.

The initial POEM description by Inoue implied an 8-cm myotomy (6 cm esophageal, 2 cm gastric).^5^ As in achalasia, the crucial site of obstruction is the LES, the somehow arbitrary extension of the myotomy is in contrast with manometric findings showing that the average normal LES extension is around 3 cm.^8^ If it is ascertained that, concerning manometric achalasia subtypes, for type III pattern a longer myotomy involving the whole extent of spastic muscle is required,^51^ from a pathophysiological perspective to expand the myotomy to healthy muscle way above the LES is debatable.^52^ Notably, our results reveal that the mean total myotomy length remains over 10 cm in non-spastic (type I and II) achalasia, where such extension finds no physiological explanation. This finding is particularly relevant in the context of recent evidence advocating for shorter POEM procedures in this setting. Nabi *et al*. conducted the first trial including type I and II achalasia patients, randomly assigning half to very short myotomy (3 cm) and half to longer myotomy (6 cm and above). At one year, both groups showed similar clinical success, AEs, and GERD rates (41% vs. 51%).^9^ Gu *et al*., in another randomized trial, compared standard myotomy (10 cm) to short myotomy (mean 5.6 cm) in type II achalasia, demonstrating comparable treatment success (96% vs. 94%). The short myotomy group exhibited reduced postoperative abnormal acid exposure (23.9% vs. 43.8%, *P* = 0.042) and shorter procedure times (31 minutes vs. 46 minutes, *P* < 0.05).^53^ More recently, Familiari *et al*. randomized 200 patients, half to long myotomy (13 cm), the other half to shorter myotomy (8 cm), finding no significant differences in clinical success or GERD rates at 6- and 24-month follow-ups, emphasizing the non-inferiority of short POEM.^10^

Our study revealed high heterogeneity (I^2^: 99.3%) concerning total myotomy length, the primary outcome of our study. This represents itself a major study finding, meaning that POEM procedural standards are highly heterogeneous across centers, but it also prompted exploration of potential contributors. Leave-one-out and subgroup analyses for study design did not explain such heterogeneity. Neither the geographic subgroup analysis could identify substantial differences between Eastern and Western countries, as we included demographically different cohorts (Asian vs. Caucasian) with inherent differences in esophageal length related to average subject height.

Moreover, our temporal trend sub-group analysis suggests a lack of significant progress towards implementation of short myotomy over time. The pooled mean myotomy length for studies conducted between 2014 and 2020 was 10.53 cm (95% CI, 10.22–10.84; I^2^ 99.1%), and no significant decrease was observed in studies in 2021–2022 (after publication of the first RCT showing comparable efficacy of a short myotomy^9^), as it still attested at 9.74 cm (95% CI, 7.95–11.54; I^2^ 99.7%), with largely overlapping 95% CIs. We noted the shortest lengths in the latest studies (2022), with a mean pooled length of 7.95 cm, possibly suggesting an only recent shift towards short myotomy adoption, yet future studies are necessary to confirm this observation. The reluctance to adopt a shorter POEM despite emerging evidence supporting its efficacy and safety highlights a gap between evolving research findings and clinical practice. Short myotomy may be beneficial as it may avoid unnecessary submucosal tunneling and myotomy, potentially reducing the risk of intraprocedural (bleeding, perforation) and long-term (GERD and ‘blown out myotomy’ [BOM], i.e. a pseudodiverticulum of the distal esophagus caused by myotomy-related wall weakening) complications.^52^^,^^54^ In conclusion, the absence of a clear trend suggests a reluctance to change in the accepted norms of myotomy length, even in the face of evolving evidence supporting short POEM, warranting further studies on factors influencing technical decisions.

Our study, while contributing substantially to the clarification of POEM current standards, acknowledges certain limitations. Almost all studies included were retrospective cohort analysis with a lack of standardization in clinical outcome assessment, despite the fact that those data were not the primary outcome of the analysis. Consequently, meta-regressions with the identification of clear correlations between different myotomy lengths with reflux symptoms, erosive esophagitis occurrence, or abnormal pH-metry results were not performed. Therefore, clinical implications based on the primary outcome (myotomy length) could not be explored, but future studies are warranted. Furthermore, inter-study heterogeneity assessed resulted relevant and non-completely explainable with leave-one-out analysis or sub-group analysis performed, probably due to substantial heterogeneity of cohorts in different studies, from baseline characteristics to outcome definitions and assessment. Nonetheless, as above stated, the heterogeneity between studies describing the same procedure for the same disease could easily reflect the substantial disagreement and lack of standardization on POEM technique and its pathophysiological downsides, strengthening *de facto* the purpose of our meta-analysis. Lastly, there may have been an inherent bias towards including studies with inappropriately ‘long’ myotomy, often exceeding 10 cm. This may have arisen because studies comparing different myotomy lengths, adopting pre-specified myotomy length, and FLIP-tailored myotomy were (in our view appropriately since the main study aim was to identify a ‘standard’ POEM length) excluded from the analysis. Consequently, our findings may not have fully represented current technical POEM trends.

In conclusion, our meta-analysis found that the standard myotomy length during POEM for achalasia in clinical practice is 10.4 cm. The persistent use of long myotomy in type I/II (non-spastic) achalasia, the high heterogeneity observed, and the reluctance in adopting short POEM despite recent evidence supporting efficacy and safety of this approach, highlight the need of procedural standardization. Consensus guidelines focusing on POEM technical details are needed.

## Supplementary Material

Supplementary_data_doae069

## Data Availability

No new data were generated or analyzed in support of this research.

## Notes

These authors contributed equally to this work. Specific author contributions: EV and AB conceived the article. EV and AB drafted the manuscript. AB, EF, and MCF performed to the systematic review. EV and AB created tables and figures. FVM, SP, FA, EVS, and SD critically reviewed the content of the paper. The final manuscript was approved by all authors. Conflicts of interest: The authors declare that they have no conflict of interest related to this paper. Financial support: None.
