# Supplementary material for: Standard length of peroral endoscopic myotomy (POEM) for achalasia: a systematic review and meta-analysis
Source: Dis Esophagus. 2024 Aug 30;37(12):doae069. doi: 10.1093/dote/doae069 (PMC11605639; doi:10.1093/dote/doae069)

**Supplementary Materials**

**Supplementary Table 1.** Preferred Reporting Items for Systematic Reviews and Meta-Analyses (PRISMA) checklist

| **Section/topic** | **#** | **Checklist item** | **Reported on page #** |
| --- | --- | --- | --- |
| **TITLE** | | |  |
| Title | 1 | Identify the report as a systematic review, meta-analysis, or both. | 1 |
| **ABSTRACT** | | |  |
| Structured summary | 2 | Provide a structured summary including, as applicable: background; objectives; data sources; study eligibility criteria, participants, and interventions; study appraisal and synthesis methods; results; limitations; conclusions and implications of key findings; systematic review registration number. | 2 |
| **INTRODUCTION** | | |  |
| Rationale | 3 | Describe the rationale for the review in the context of what is already known. | 3-4 |
| Objectives | 4 | Provide an explicit statement of questions being addressed with reference to participants, interventions, comparisons, outcomes, and study design (PICOS). | 4 |
| **METHODS** | | |  |
| Protocol and registration | 5 | Indicate if a review protocol exists, if and where it can be accessed (e.g., Web address), and, if available, provide registration information including registration number. | 4 |
| Eligibility criteria | 6 | Specify study characteristics (e.g., PICOS, length of follow-up) and report characteristics (e.g., years considered, language, publication status) used as criteria for eligibility, giving rationale. | 5 |
| Information sources | 7 | Describe all information sources (e.g., databases with dates of coverage, contact with study authors to identify additional studies) in the search and date last searched. | 4 |
| Search | 8 | Present full electronic search strategy for at least one database, including any limits used, such that it could be repeated. | 4, Supplementary table 2 |
| Study selection | 9 | State the process for selecting studies (i.e., screening, eligibility, included in systematic review, and, if applicable, included in the meta-analysis). | 5, Figure 1 |
| Data collection process | 10 | Describe method of data extraction from reports (e.g., piloted forms, independently, in duplicate) and any processes for obtaining and confirming data from investigators. | 4 |
| Data items | 11 | List and define all variables for which data were sought (e.g., PICOS, funding sources) and any assumptions and simplifications made. | 4 |
| Risk of bias in individual studies | 12 | Describe methods used for assessing risk of bias of individual studies (including specification of whether this was done at the study or outcome level), and how this information is to be used in any data synthesis. | 6 |
| Summary measures | 13 | State the principal summary measures (e.g., risk ratio, difference in means). | 7 |
| Synthesis of results | 14 | Describe the methods of handling data and combining results of studies, if done, including measures of consistency (e.g., I^2^) for each meta-analysis. | 7 |
| Risk of bias across studies | 15 | Specify any assessment of risk of bias that may affect the cumulative evidence (e.g., publication bias, selective reporting within studies). | 6 |
| Additional analyses | 16 | Describe methods of additional analyses (e.g., sensitivity or subgroup analyses, meta-regression), if done, indicating which were pre-specified. | 7 |
| **RESULTS** | | |  |
| Study selection | 17 | Give numbers of studies screened, assessed for eligibility, and included in the review, with reasons for exclusions at each stage, ideally with a flow diagram. | Figure 1 |
| Study characteristics | 18 | For each study, present characteristics for which data were extracted (e.g., study size, PICOS, follow-up period) and provide the citations. | 8,  Table 1-3, Supplementary Table 4 |
| Risk of bias within studies | 19 | Present data on risk of bias of each study and, if available, any outcome level assessment (see item 12). | 8-9, Supplementary Table 3 |
| Results of individual studies | 20 | For all outcomes considered (benefits or harms), present, for each study: (a) simple summary data for each intervention group (b) effect estimates and confidence intervals, ideally with a forest plot. | 9-11, Figures 2-6, Supplementary Figures 2-6 |
| Synthesis of results | 21 | Present results of each meta-analysis done, including confidence intervals and measures of consistency. | 9-11, Figures 2-6, Supplementary Figures 2-6 |
| Risk of bias across studies | 22 | Present results of any assessment of risk of bias across studies (see Item 15). | 8-9, Supplementary figure 1 and Supplementary Table 3 |
| Additional analysis | 23 | Give results of additional analyses, if done (e.g., sensitivity or subgroup analyses, meta-regression [see Item 16]). | 10-11, Figure 5,6,7 Supplementary Figures 2-6 |
| **DISCUSSION** | | |  |
| Summary of evidence | 24 | Summarize the main findings including the strength of evidence for each main outcome; consider their relevance to key groups (e.g., healthcare providers, users, and policy makers). | 11-13 |
| Limitations | 25 | Discuss limitations at study and outcome level (e.g., risk of bias), and at review-level (e.g., incomplete retrieval of identified research, reporting bias). | 13 |
| Conclusions | 26 | Provide a general interpretation of the results in the context of other evidence, and implications for future research. | 13 |
| **FUNDING** | | |  |
| Funding | 27 | Describe sources of funding for the systematic review and other support (e.g., supply of data); role of funders for the systematic review. | 14 |

**Supplementary Table 2.** Detailed search strategy for systematic review

| Name of database | Time span | Search strategy |
| --- | --- | --- |
|  |  |  |
| **PubMed/MEDLINE** | 2008- Apr 2023 | ('peroral endoscopic myotomy'/exp OR 'peroral endoscopic myotomy' OR (peroral AND endoscopic AND ('myotomy'/exp OR myotomy)) OR 'endoscopic myotomy'/exp OR 'endoscopic myotomy' OR (endoscopic AND ('myotomy'/exp OR myotomy)) OR 'peroral myotomy' OR (peroral AND ('myotomy'/exp OR myotomy)) OR 'endosc myoto' OR (endosc AND myoto) OR poem OR 'poem procedure' OR (poem AND ('procedure'/exp OR procedure)) OR 'poem procedures' OR (poem AND ('procedures'/exp OR procedures)) OR 'endoscopic esophageal myotomy' OR (endoscopic AND esophageal AND ('myotomy'/exp OR myotomy)) OR 'peroral esophageal myotomy'/exp OR 'peroral esophageal myotomy' OR (peroral AND esophageal AND ('myotomy'/exp OR myotomy)) OR 'endosc esoph myot' OR (endosc AND esoph AND myot)) AND ('achalasia'/exp OR achalasia) |
| **EMBASE**  **Scopus**  **Cochrane** | 2008- Apr 2023  2008- Apr 2023  2008- Apr 2023 | ('peroral endoscopic myotomy'/exp OR 'peroral endoscopic myotomy' OR (peroral AND endoscopic AND ('myotomy'/exp OR myotomy)) OR 'endoscopic myotomy'/exp OR 'endoscopic myotomy' OR (endoscopic AND ('myotomy'/exp OR myotomy)) OR 'peroral myotomy' OR (peroral AND ('myotomy'/exp OR myotomy)) OR 'endosc myoto' OR (endosc AND myoto) OR poem OR 'poem procedure' OR (poem AND ('procedure'/exp OR procedure)) OR 'poem procedures' OR (poem AND ('procedures'/exp OR procedures)) OR 'endoscopic esophageal myotomy' OR (endoscopic AND esophageal AND ('myotomy'/exp OR myotomy)) OR 'peroral esophageal myotomy'/exp OR 'peroral esophageal myotomy' OR (peroral AND esophageal AND ('myotomy'/exp OR myotomy)) OR 'endosc esoph myot' OR (endosc AND esoph AND myot)) AND ('achalasia'/exp OR achalasia)  ('peroral endoscopic myotomy'/exp OR 'peroral endoscopic myotomy' OR (peroral AND endoscopic AND ('myotomy'/exp OR myotomy)) OR 'endoscopic myotomy'/exp OR 'endoscopic myotomy' OR (endoscopic AND ('myotomy'/exp OR myotomy)) OR 'peroral myotomy' OR (peroral AND ('myotomy'/exp OR myotomy)) OR 'endosc myoto' OR (endosc AND myoto) OR poem OR 'poem procedure' OR (poem AND ('procedure'/exp OR procedure)) OR 'poem procedures' OR (poem AND ('procedures'/exp OR procedures)) OR 'endoscopic esophageal myotomy' OR (endoscopic AND esophageal AND ('myotomy'/exp OR myotomy)) OR 'peroral esophageal myotomy'/exp OR 'peroral esophageal myotomy' OR (peroral AND esophageal AND ('myotomy'/exp OR myotomy)) OR 'endosc esoph myot' OR (endosc AND esoph AND myot)) AND ('achalasia'/exp OR achalasia)  (Peroral endoscopic myotomy OR POEM OR peroral myotomy OR endoscopic myotomy OR esophageal myotomy OR endoscopic esophageal myotomy) AND achalasia |

**Supplementary Table 3.** Newcastle Ottawa Scale (NOS) assessment for Cohort studies.

| Studies | Selection | | | | Comparability | Outcome | | | Total stars |
| --- | --- | --- | --- | --- | --- | --- | --- | --- | --- |
|  |  |  |  |  |  |  |  |  |  |
|  | 1.Representativeness of the exposed cohort | 2. Selection of the unexposed cohort | 3.Ascertainment of exposure | 4.Demonstration that outcome of interest was not present at start of study | 5. Comparability of cohorts on the basis of the design or analysis | 6. Assessment of outcome | 7. Was follow-up long enough for outcomes to occur | 8. Adequacy of follow up of cohorts |  |
|  |  |  |  |  |  |  |  |  |  |
| **Chang J et al.** | ☆ | - | - | ☆ | ☆ | - | ☆ | ☆ | 5 |
| **Chen et al.** | ☆ | - | - | ☆ | - | ☆ | - | - | 3 |
| **Arshava E,V et al.** | ☆ | - | - | ☆ | - | - | - | ☆ | 3 |
| **De Pascale S et al.** | ☆ | - | - | ☆ | ☆ | ☆ | - | ☆ | 5 |
| **DeWitt J,M et al.** | ☆ | - | - | ☆ | - | ☆ | ☆ | - | 4 |
| **Qiu S et al.** | ☆ | - | ☆ | - | ☆ | ☆ | - | ☆ | 5 |
| **Guo H et al.** | ☆ | - | ☆ | ☆ | ☆ | ☆ | - | ☆ | 6 |
| **Karyampudi A et al.** | ☆ | - | - | ☆ | - | ☆ | - | - | 3 |
| **Liu HY et al.** | ☆ | - | ☆ | - | ☆ | ☆ | ☆ | ☆ | 6 |
| **Khashab M et al.** | ☆ | ☆ | ☆ | ☆ | ☆ | ☆ | ☆ | - | 7 |
| **Yang et al.** | ☆ | - | - | ☆ | - | ☆ | - | - | 3 |
| **Sanaka et al.** | ☆ | - | - | ☆ | - | ☆ | - | - | 3 |
| **Raja et al.** | ☆ | - | - | ☆ | - | ☆ | - | - | 3 |
| **Nast et al.** | ☆ | - | - | ☆ | - | ☆ | - | - | 3 |
| **Hu JW et al.** | ☆ | ☆ | - | ☆ | ☆ | ☆ | ☆ | - | 6 |
| **Dacha S et al.** | ☆ | - | ☆ | ☆ | ☆ | - | ☆ | ☆ | 6 |
| **Teh JL et al.** | ☆ | - | - | ☆ | ☆ | ☆ | ☆ | - | 5 |
| **Evensen H et al.** | ☆ | - | - | ☆ | - | ☆ | - | - | 3 |
| **Ichkhanian Y et al.** | ☆ | ☆ | - | ☆ | ☆ | ☆ | - | ☆ | 6 |
| **Mondragòn H et al.** | ☆ | - | - | ☆ | - | ☆ | - | ☆ | 4 |
| **Xu Y et al.** | ☆ | - | - | ☆ | - | ☆ | - | - | 3 |
| **Wang N, et al.** | ☆ | - | - | ☆ | ☆ | - | ☆ | ☆ | 5 |
| **Farias GFA et al.** | ☆ | - | - | ☆ | - | ☆ | - | ☆ | 4 |
| **Tang X et al.** | ☆ | - | - | ☆ | - | ☆ | - | ☆ | 4 |
| **Werner et al.** | ☆ | ☆ | ☆ | ☆ | ☆ | ☆ | - | ☆ | 7 |

**Supplementary Table 4.** Myotomy lengths in achalasia manometric subtypes (I and II versus spastic-type III)

| **Author** | **Year** | **Achalasia type I** | **Achalasia type II** | **Achalasia type III (spastic type)** |
| --- | --- | --- | --- | --- |
|  |  | **Total myotomy length, cm (SD)** | **Total myotomy length, cm (SD)** | **Total myotomy length, cm (SD)** |
| Chang J et al | 2019 | - | 9.5 (1.6) | - |
| Chen et al, | 2014 | 9.6 (1.4) | - | 9.6 (1.5) |
| Arshava E, V et al, | 2018 | - | - | - |
| De Pascale S, | 2017 | - | 8.9 (1.8) | - |
| DeWitt J, M, et al, | 2022 | 10 (1.9) | - | 15 (4.4) |
| Qiu S, et al, | 2021 | - | 10.6 (2.7) | - |
| Guo H, et al, | 2017 | 10.6 (2.7) | - | 10.9 (2.1) |
| Karyampudi A, et al, | 2020 | - | - | - |
| Liu HY et al, 1 | 2019 | - | - | - |
| Liu HY et al, 2 | 2019 | - | 10.9 (0.2) |  |
| Kashab M, et al, 1 | 2020 | 9.7 (0.2) | 10.9 (0.2) | 13 (0.9) |
| Kashab M, et al, 2 | 2020 | 10.5 (1.15) | - | 15.3 (1.7) |
| Yang et al, | 2015 | - | - | - |
| Sanaka et al, 1 | 2020 | - | - | - |
| Sanaka et al, 2 | 2020 | - | - | - |
| Raja et al, | 2018 | - | - | - |
| Nast et al, | 2018 | - | - | - |
| Hu JW, et al, | 2014 | - | - | - |
| Dacha S, et al, | 2018 | - | - | - |
| Teh JL, et al, | 2021 | - | - | - |
| Evensen H, et al, | 2021 | - | - | - |
| Ichkhanian Y, et al, 1 | 2020 | - | - | - |
| Ichkhanian Y, et al, 2 | 2020 | - | 11.2 (2.2) | - |
| Mondragòn H, et al, | 2019 | 9 (1.7) | - | 19.2 (0.4) |
| Xu Y, et al, 1 | 2020 | - | - | - |
| Xu Y, et al, 2 | 2020 | - | - | - |

**Supplementary table 5.** Main outcomes after POEM

| **Author** | **Year** | **Clinical success, n (%)** | **Clinical success definition** | **Mean post-Eckardt score (SD, or range, IQR)** | **GERD symptoms, n (%)** | **GERD symptoms definition** | **Erosive Esophagitis, n (%)** | **PPI use, n (%)** | **Abnormal pH-metry** | **Adverse events, n (%)** |
| --- | --- | --- | --- | --- | --- | --- | --- | --- | --- | --- |
| Chang et al. | 2019 | NA | NA | NA | 36 (18.5) | GERD-Q | NA | NA | NA | NA |
| Chen et al. | 2014 | NA | NA | 1.3 (1.2) | NA | NA | NA | NA | NA | NA |
| Arshava et al. | 2018 | 29 (93.5) | Symptoms relieve | 73.8 (24.2) | NA | NA | NA | NA | NA | 1 (3.2) |
| De Pascale et al. | 2017 | NA | NA | Median 1.4 [0-8] | 4 (12.5) | NA | 8 (25) | 4 (12.5) | 5 (15.6) | 4 (12.5) |
| DeWitt et al. | 2022 | 85 (97.7) | ES <3 | Median 63.4 [32-114] | NA | NA | NA | NA | NA | NA |
| Qiu et al. | 2021 | 94 (93.1) | ES <3 | NA | 27 (26.7) | NA | NA | NA | NA | 10 (8.9) |
| Guo et al. | 2017 | 59 (88.1) | Symptoms relapse | 1.0 (NA) | 9 (13.4) | NA | NA | NA | NA | 19 (28.3) |
| Karyampudi et al. | 2020 | NA | NA | 1.9 (1.7) | 33 (66) | NA | 22 (64.7) | NA | 34 (68) | NA |
| Liu et al. 1 | 2019 | 79 (92.9) | NA | Median 0 [0-1] | 29 (49.1) | NA | NA | NA | NA | 4 (2.8) |
| Liu et al. 2 | 2019 | 163 (92.6) | NA | 0.6 (1.3) | 56 (31.8) | NA | NA | NA | NA | 6 (2.1) |
| Khashab et al. 1 | 2020 | 64 (90.1) | ES <3 | 0.6 (1.2) | 29 (49.1) | DeMeester | NA | 19 (28.3) | 29 (49.1) | 8 (11.2) |
| Khashab et al. 2 | 2020 | 70 (90.9) | ES <3 | Median 0 [0-1] | 25 (41.7) | DeMeester | NA | 20 (27.8) | 25 (41.7) | 7 (9.1) |
| Yang et al. | 2015 | 40 (90.9) | ES <3 | Median 0 [0-1] | NA | NA | NA | NA | NA | 5 (9.6) |
| Sanaka et al. 1 | 2020 | NA | NA | 1.1 (NA) | 17 (19.8) | NA | NA | NA | NA | 5 (5.3) |
| Sanaka et al. 2 | 2020 | NA | NA | 1 (NA) | 6 (11.8) | NA | NA | NA | NA | 1 (1.8) |
| Raja et al. | 2018 | NA | NA | 1 (NA) | 49 (49.5) | DeMeester | NA | 49 (49.5) | NA | 41 (26.9) |
| Nast et al. | 2018 | NA | NA | Median 0 [0-1] | NA | NA | NA | NA | NA | 22 (19.3) |
| Hu et al. | 2014 | 30 (96.8) | ES <3 | NA | 7 (21.9) | GERD-Q | 6 (18.7) | NA | NA | 9 (28.1) |
| Dacha et al. | 2018 | 58 (93.5) | ES <3 | Median 1.4 [0-5] | 8 (12.9) | NA | NA | 8 (12.9) | NA | 4 (6.4) |
| Teh et al. | 2021 | 39 (88.6) | ES <3 | 2.6 (1.2) | 25 (43.1) | NA | 24 (60) | NA | 14 (56) | 16 (27.5) |
| Evensen et al. | 2021 | 46 (92) | ES <3 | 0.8 (1.1) | 15 (36.6) | NA | 24 (48.9) | NA | 13 (27.7) | 25 (50) |
| Ichkhanian et al. 1 | 2020 | 46 (85.2) | ES <3 | Median 2 [0-3] | 13 (24.1) | GERD-Q | NA | 10 (18.5) | NA | NA |
| Ichkhanian et al. 2 | 2020 | 45 (78.9) | ES <3 | NA | 12 (21.1) | GERD-Q | NA | 7 (12.3) | NA | NA |
| Mondragòn et al. | 2019 | 45 (100) | (IRP) < 15 mmHg, ES < 3 | NA | 8 (17.8) | NA | 9 (20) | NA | 24 (53.3) | 20 (44.4) |
| Xu et al. 1 | 2020 | 33 (86.8) | ES <3 | Median 1 [1-3] | 8 (21.1) | GERD-Q | NA | NA | NA | 18 (47.3) |
| Xu et al. 2 | 2020 | 35 (87.5) | ES <3 | Median 1 [0-3] | 4 (10) | GERD-Q | NA | NA | NA | 21 (52.2) |
| Wang et al. 1 | 2022 | 515 (88.9) | ES <3 | Median 1 [1-2.7] | 68 (11.8) | NA | NA | NA | NA | 53 (9.1) |
| Wang et al. 2 | 2022 | 102 (82.9) | ES <3 | Median 1.2 [0-4] | 15 (12.2) | NA | NA | NA | NA | 26 (21.1) |
| Farias et al. | 2019 | 29 (93.5) | ES <3 | Median 1.4 [0-5] | NA | NA | 12 (38.7) | NA | NA | 4 (12.9) |
| Tang et al. | 2017 | 80 (89.8) | ES <3 | Median 0 [0-2] | 11 (22) | NA | NA | NA | NA | 18 (18.9) |
| Werner et al. | 2019 | 89 (82.4) | NA | 1.6 (1.4) | 42 (38.9) | NA | NA | NA | NA | 12 (10.7) |

ES: Eckardt Score; GERD: Gastroesophageal Reflux Disease; IQR: Interquartile Range; IRP: Integral Relaxing Pressure; PPI: Proton Pump Inhibitors; SD: Standard Deviation

**Supplementary Table 6.** Myotomy lengths in different western and eastern countries

| **Western/Eastern** | **Country** | **Author** | **Year** | **Total myotomy length, cm (SD)** | **Pooled myotomy length, cm (95% CI)** |
| --- | --- | --- | --- | --- | --- |
| Eastern countries | China | Chen et al. | 2014 | 9.5 (1.5) | 9.8 (8.6-11.1; I^2^ 99.5%) |
|  |  | Qiu S et al. | 2021 | 11.2 (0.5) |  |
|  |  | Guo H et al. | 2017 | 10.7 (2.3) |  |
|  |  | Liu HY et al, 1 | 2019 | 10.6 (1.8) |  |
|  |  | Liu HY et al, 2 | 2019 | 10.2 (1.8) |  |
|  |  | Hu JW. et al. | 2014 | 10.3 (1.7) |  |
|  |  | Xu Y et al. 1 | 2020 | 10.9 (2.0) |  |
|  |  | Xu Y et al. 2 | 2020 | 11 (2.5) |  |
|  |  | Wang N et al. 1 | 2022 | 7.1 (1.9) |  |
|  |  | Wang N et al. 2 | 2022 | 7.1 (4.1) |  |
|  |  | Tang X et al. | 2017 | 9.9 (3.6) |  |
|  | Korea | Chang J et al. | 2019 | 8.96 (2.2) | - |
|  | India | Karyampudi A et al. | 2020 | 11.7 (3.8) | - |
|  | Singapore | Teh JL et al. | 2021 | 11.8 (2.4) | - |
| Western countries | USA | Arshava E V et al. | 2018 | 12.7 (1.4) |  |
|  |  | DeWitt J M, et al. | 2022 | 9.7 (2.9) | 9.99 (9.4-10.6, I^2^ 99.1%) |
|  |  | Yang et al. | 2015 | 13.1 (2.4) |  |
|  |  | Sanaka et al. 1 | 2020 | 9.0 (0.6) |  |
|  |  | Sanaka et al. 2 | 2020 | 9.5 (0.5) |  |
|  |  | Raja et al. | 2018 | 9.0 (0.3) |  |
|  |  | Dacha S et al. | 2018 | 7.5 (0.8) |  |
|  | Italy | De Pascale S. | 2017 | 11.7 (0.8) |  |
|  | Germany | Nast et al. | 2018 | 10.9 (3.7) |  |
|  | Norway | Evensen H, et al. | 2021 | 11.5 (0.6) |  |
|  | Messico | Mondragòn H et al. | 2019 | 13 (3.5) |  |
|  | Brazil | Farias GFA et al. 2 | 2019 | 11.3 (1.2) |  |

**Supplementary figure 1.** Funnel plot for primary outcome (total myotomy length) for the included studies


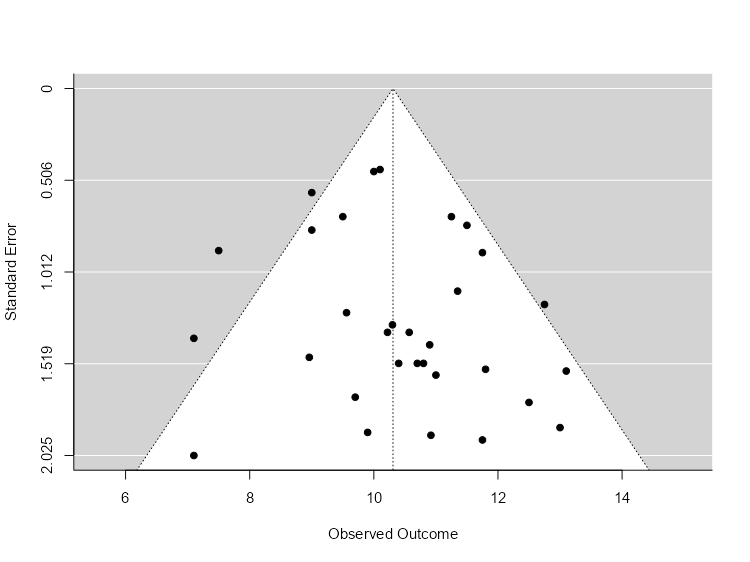


**Supplementary Figure 2.** Pooled mean esophageal (A) and gastric (B) myotomy length

**
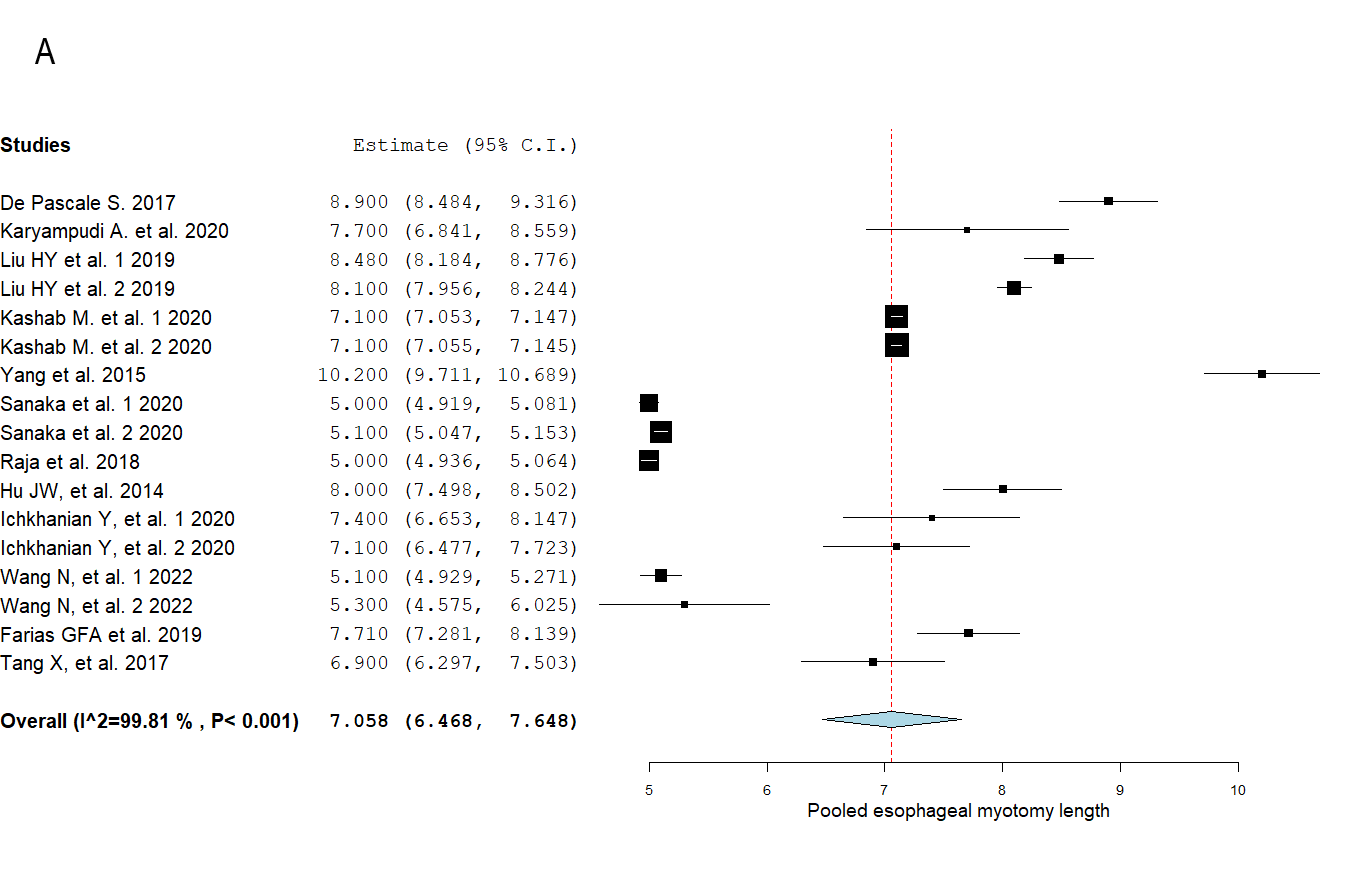

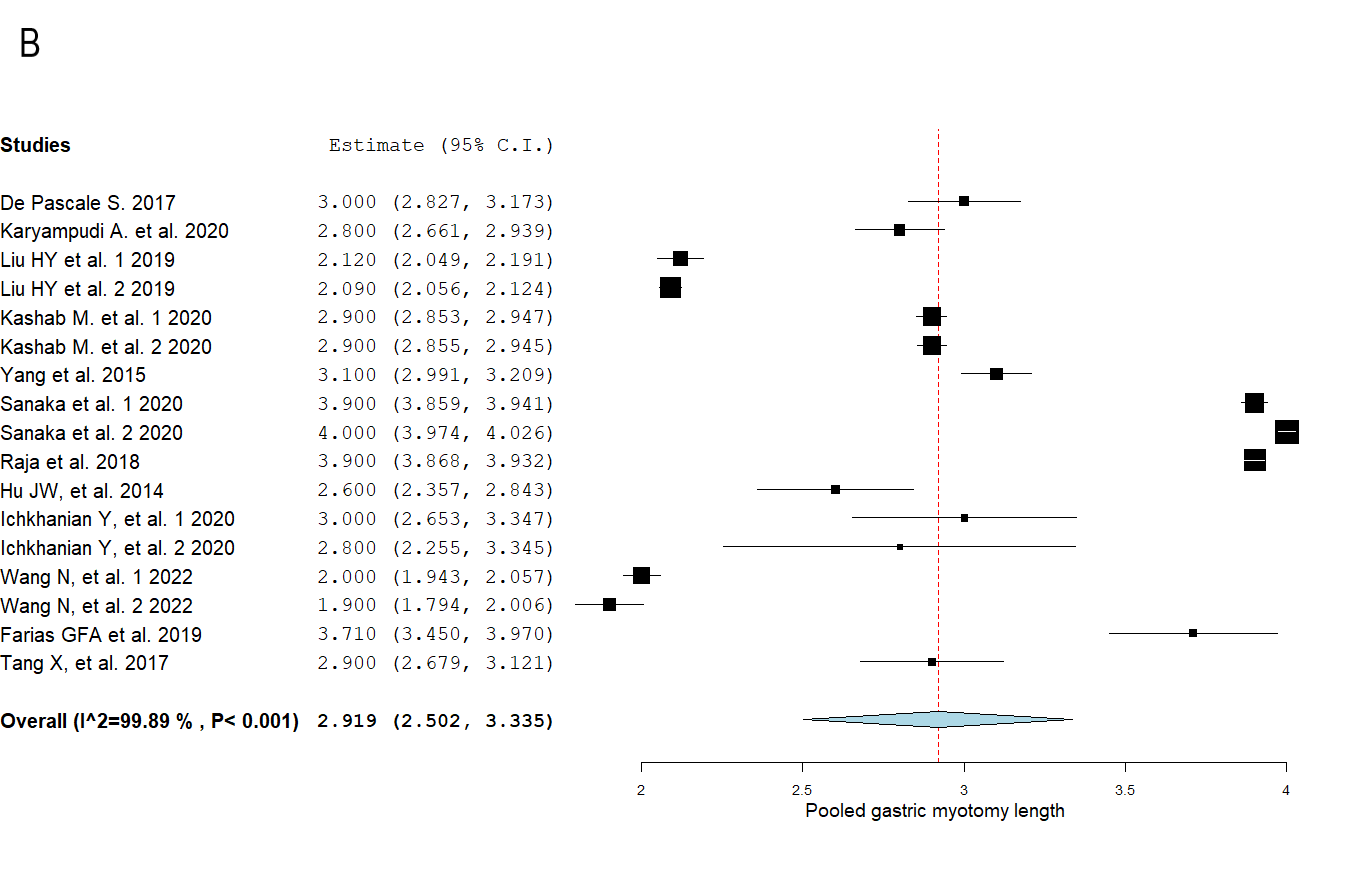
**

**Supplementary figure 3.** Leave-one-out analysis of primary outcome, pooled mean total myotomy length

**
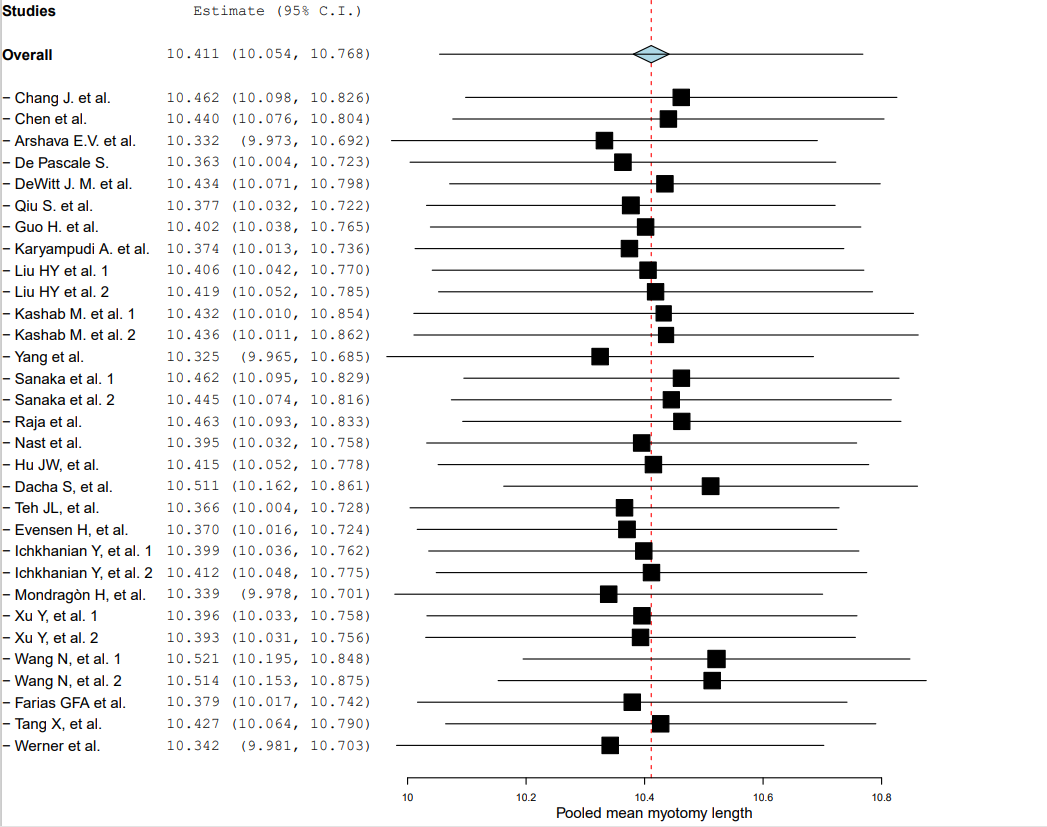
**

**Supplementary figure 4.** Pooled mean myotomy length in achalasia sub-type A) I, B) II, and C) III

A)


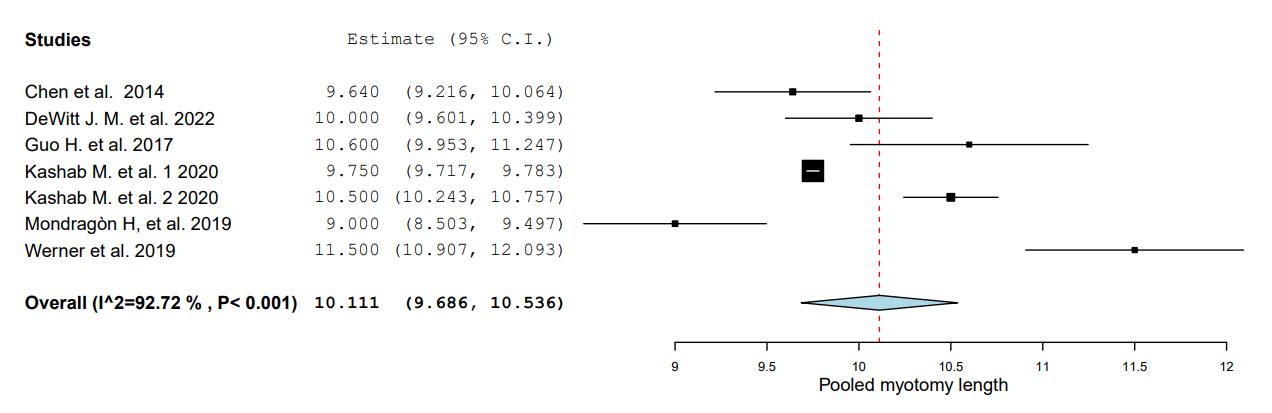


B)
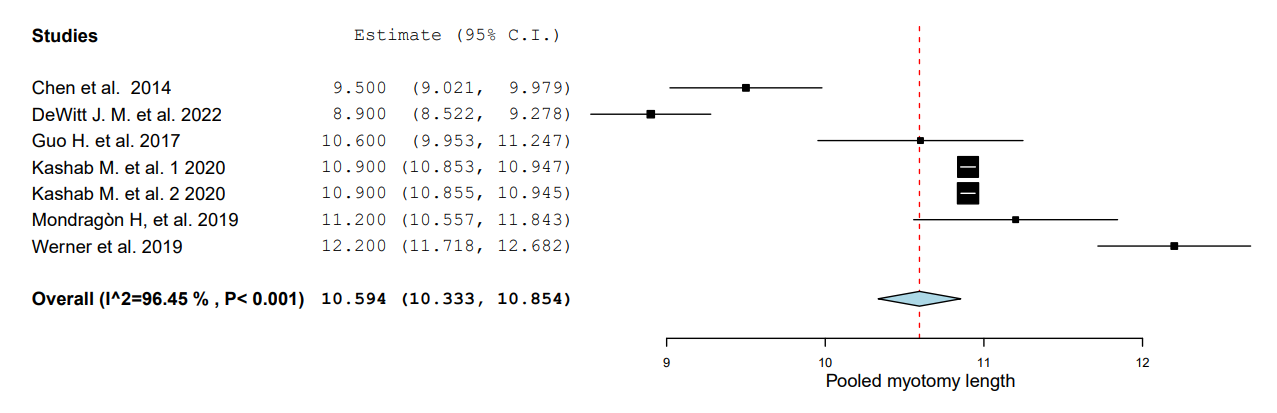


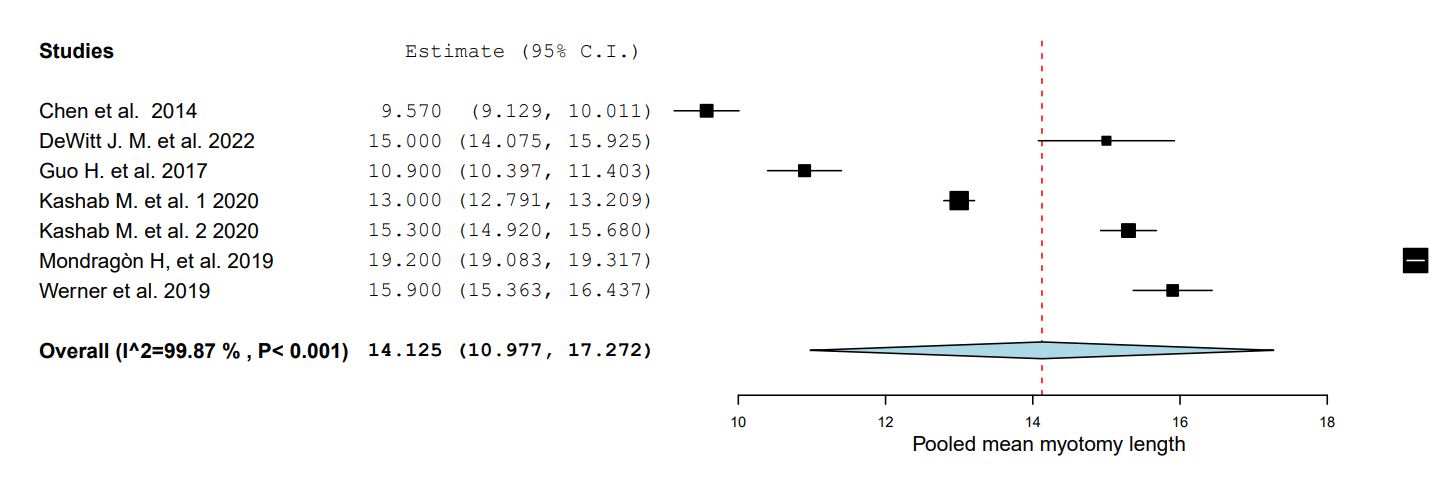
C)

**Supplementary Figure 5.** Pooled clinical success rate


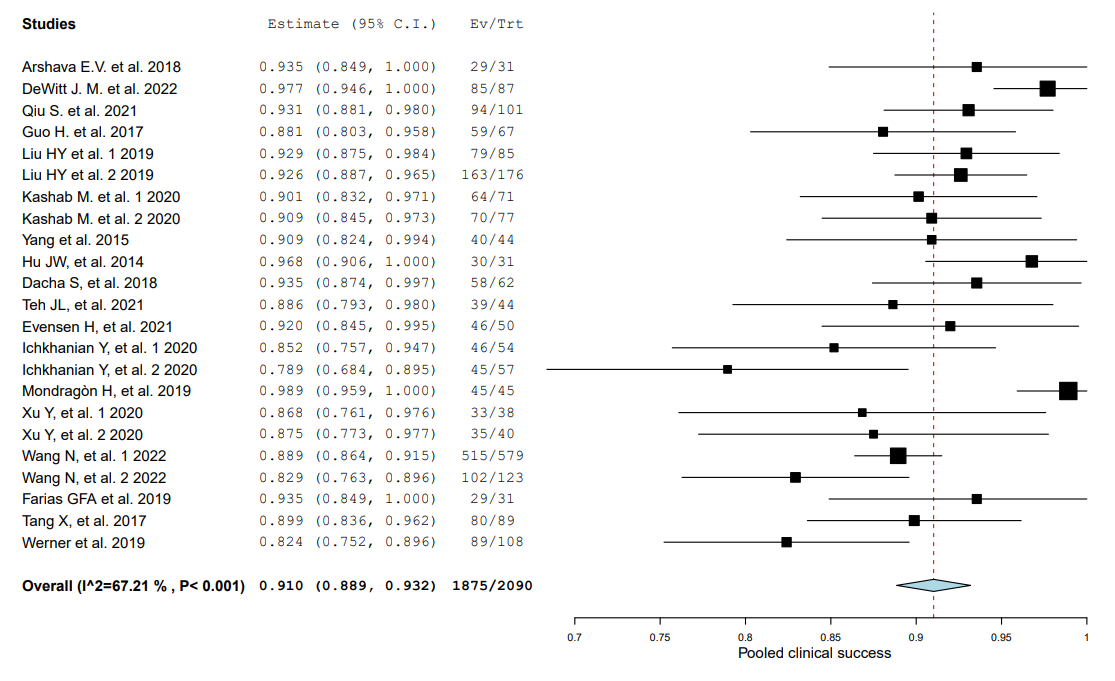


**Supplementary Figure 6.** Pooled reflux symptoms rate


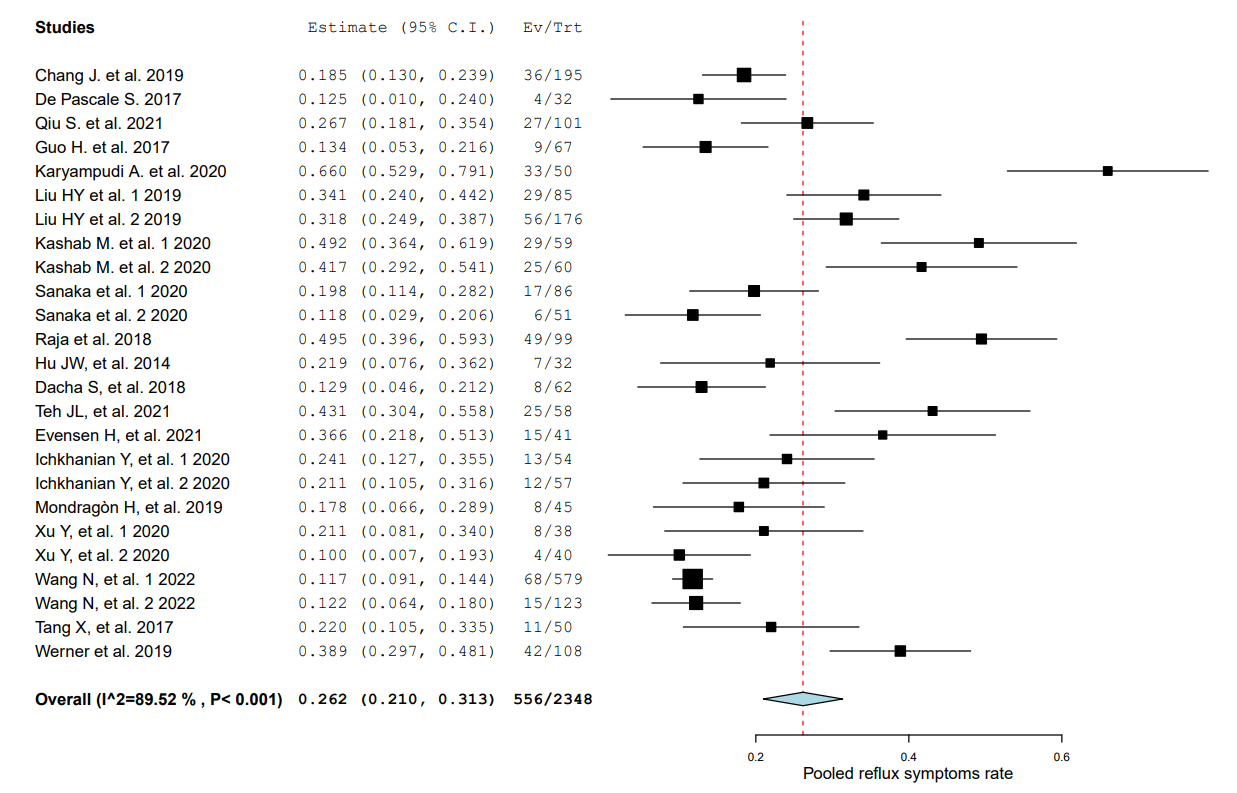


**Supplementary Figure 7.** Pooled A) total AEs and B) severe AEs rates

A)


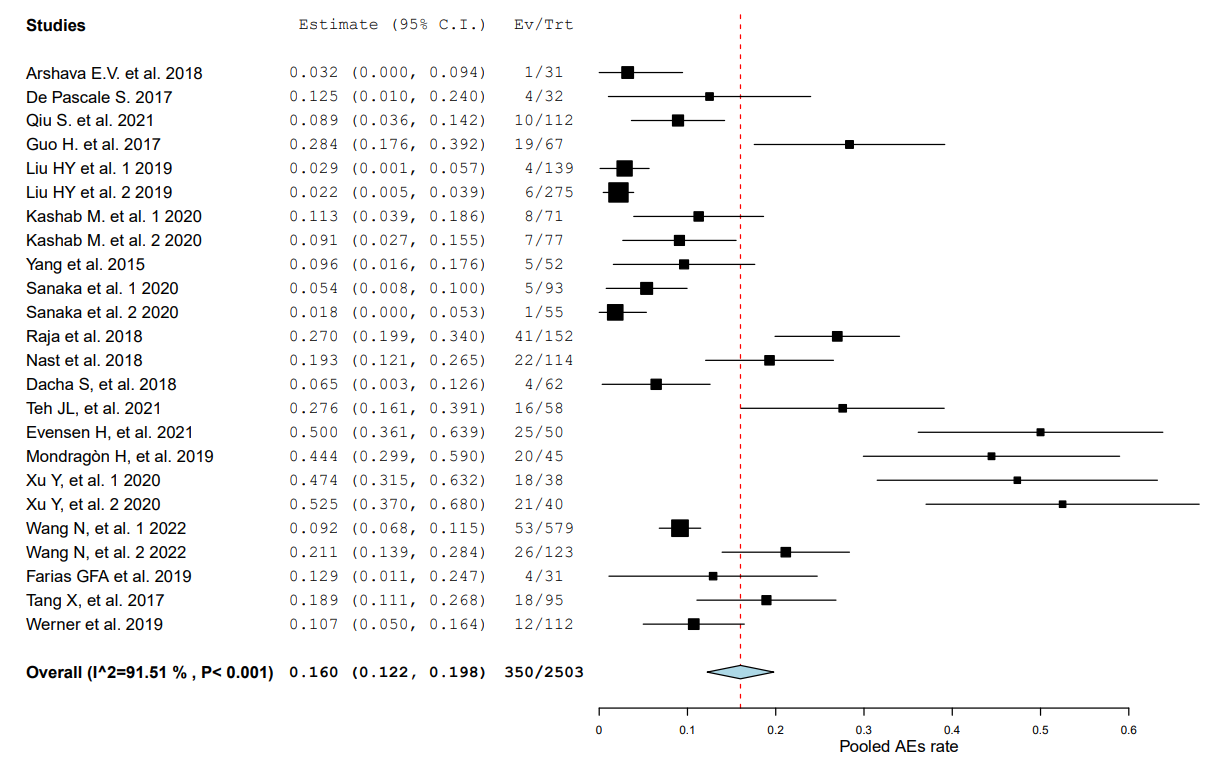


B)


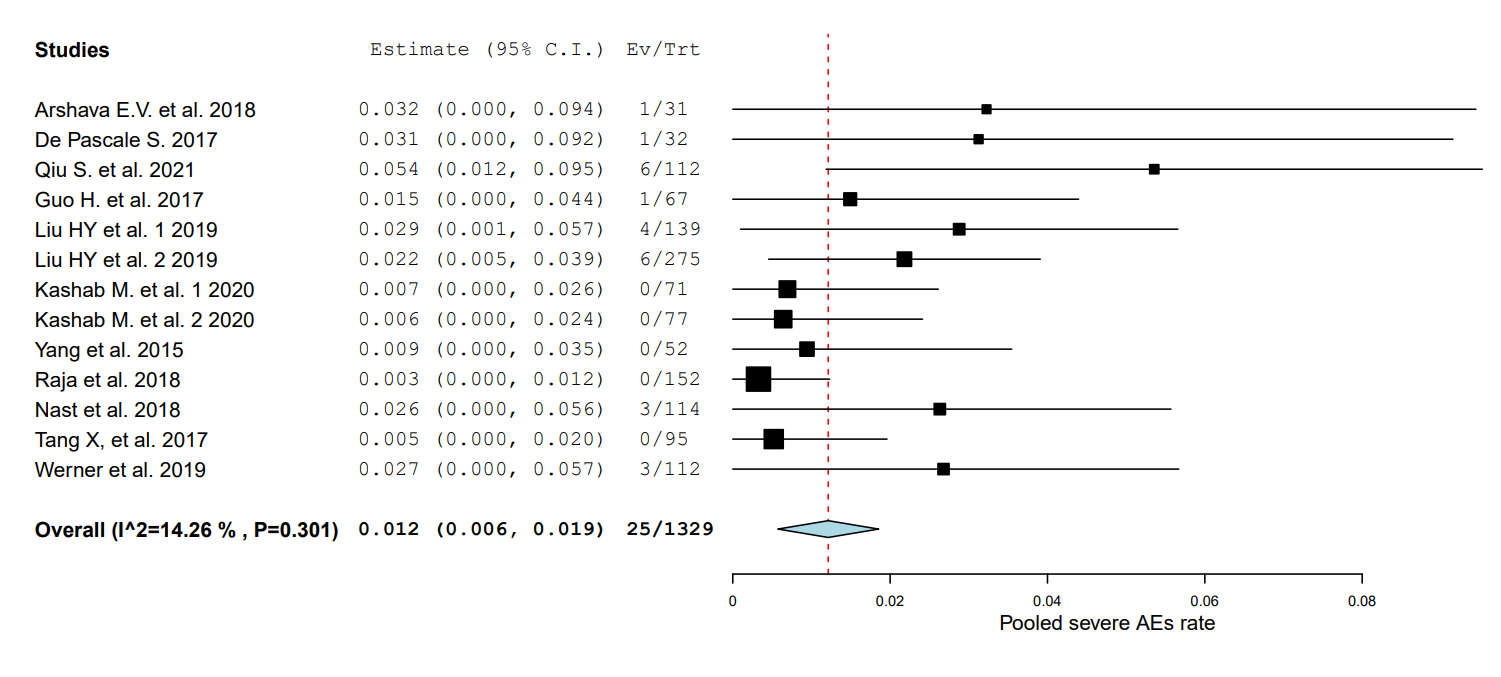


**Supplementary Figure 8.** Pooled myotomy length in A) years between 2014-2020 and B) 2021-2022
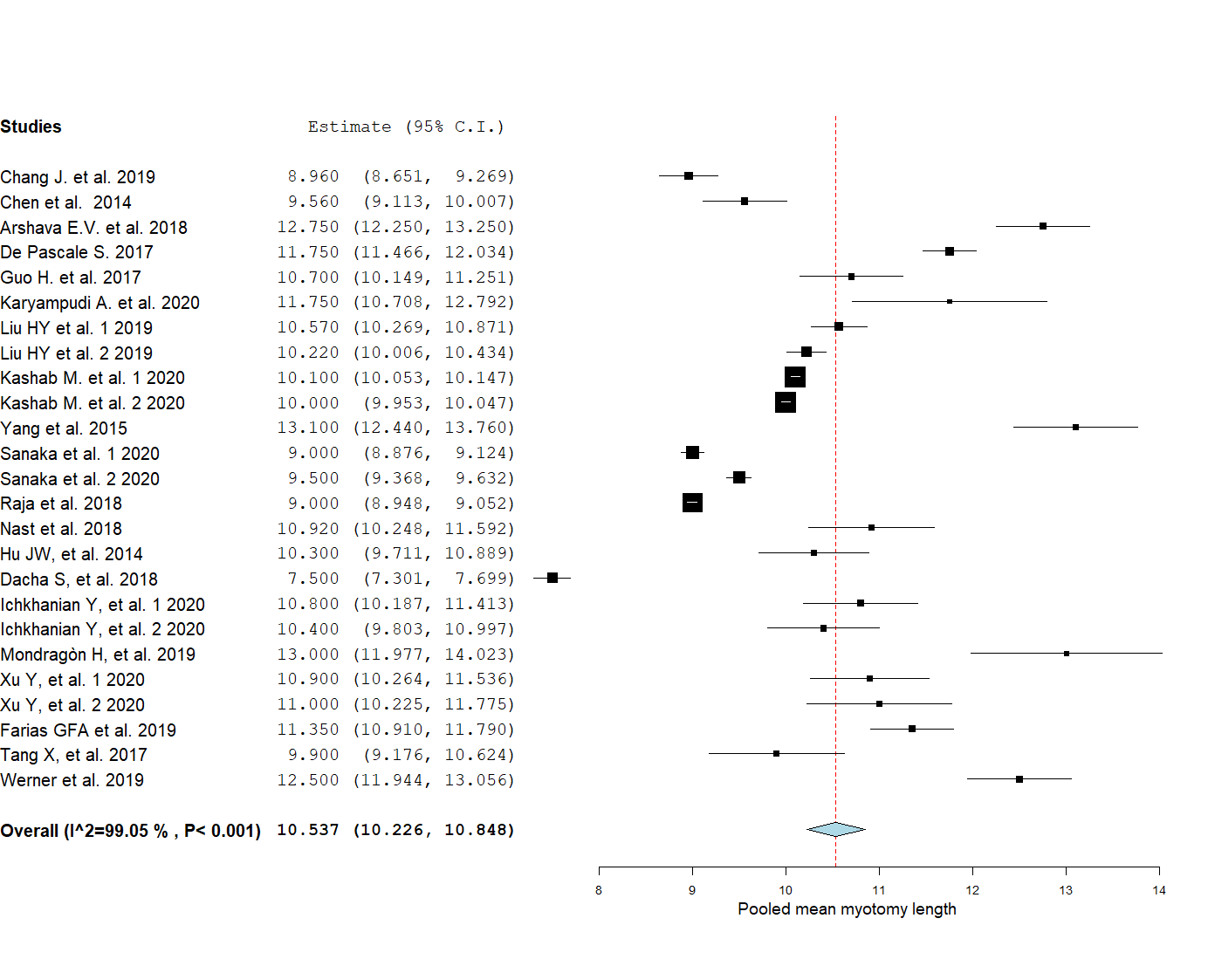

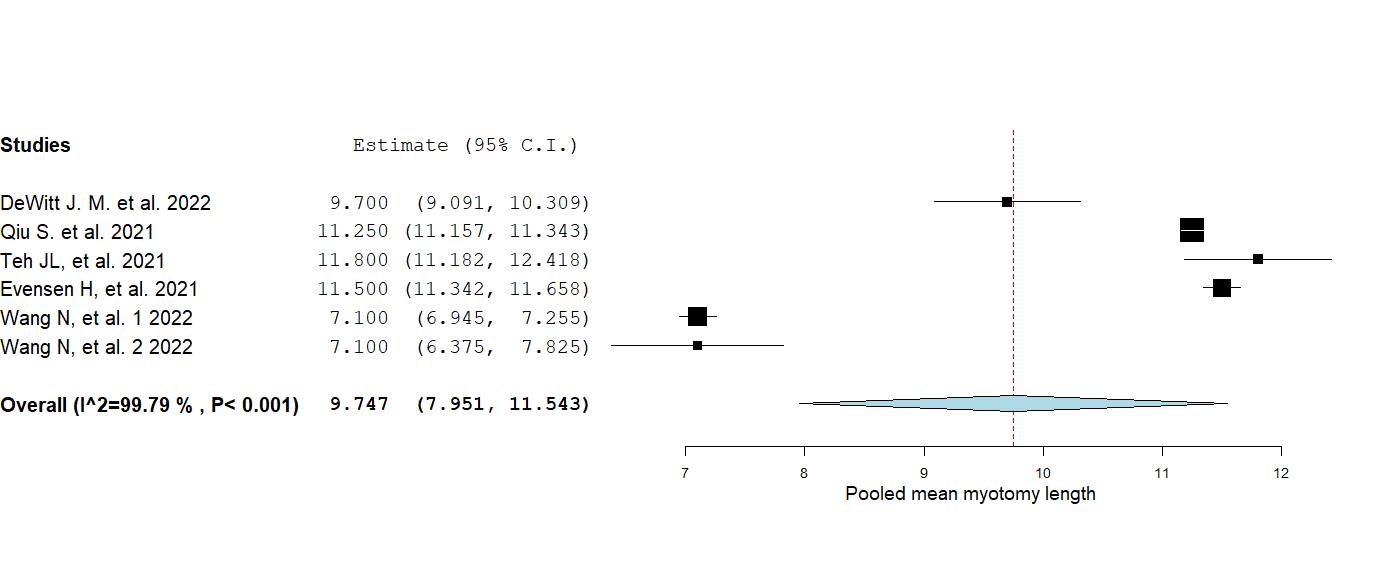

Supplement: Supplementary_data_doae069 [file supplementary_data_doae069.zip › Supplementary_Materials (1).docx]
