# Supplementary figures and images for: Standard length of peroral endoscopic myotomy (POEM) for achalasia: a systematic review and meta-analysis
Source: Dis Esophagus. 2024 Aug 30;37(12):doae069. doi: 10.1093/dote/doae069 (PMC11605639; doi:10.1093/dote/doae069)

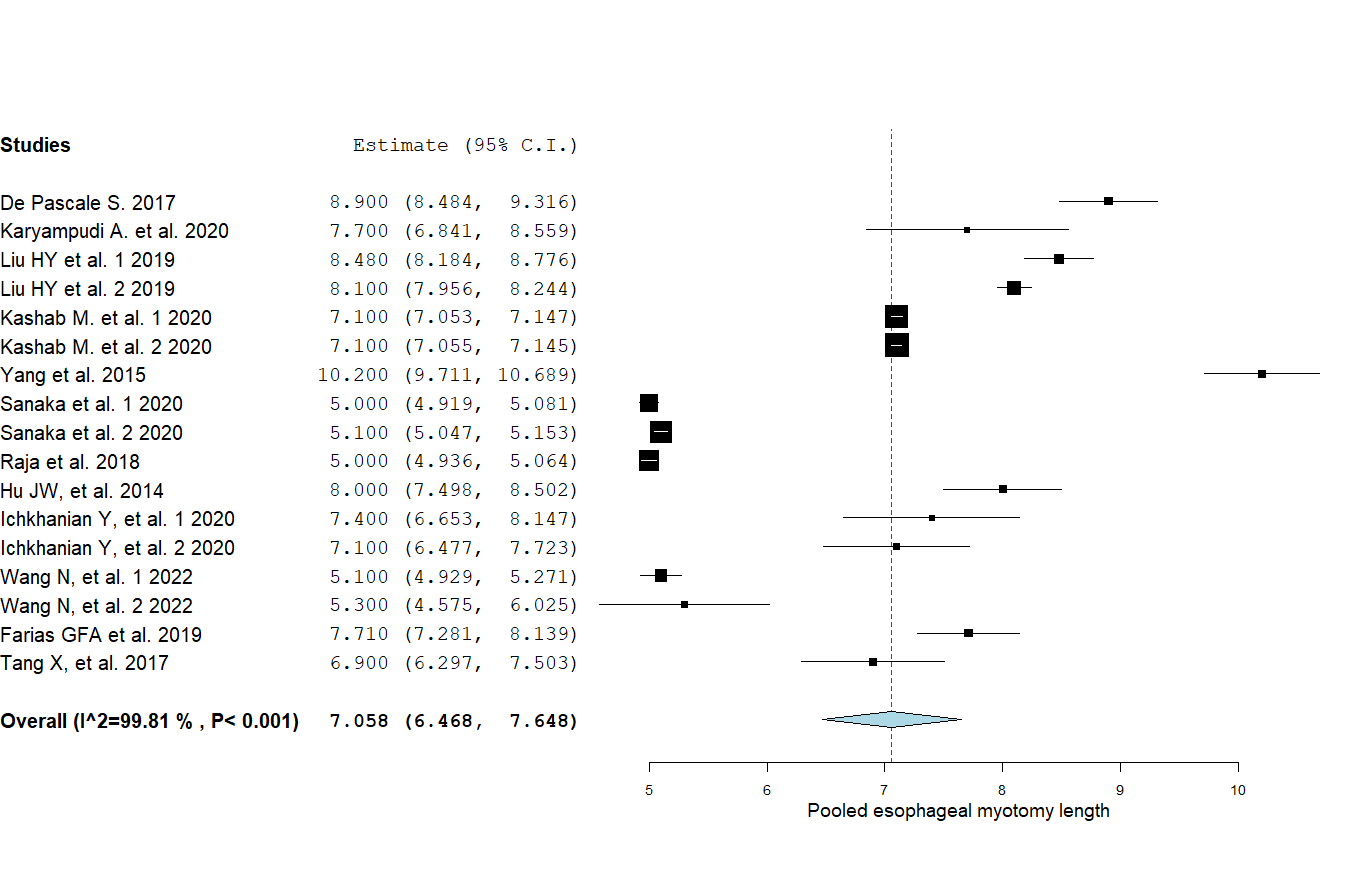

Supplement: Supplementary_data_doae069 [file supplementary_data_doae069.zip › Supplementary Figure 2A.png]

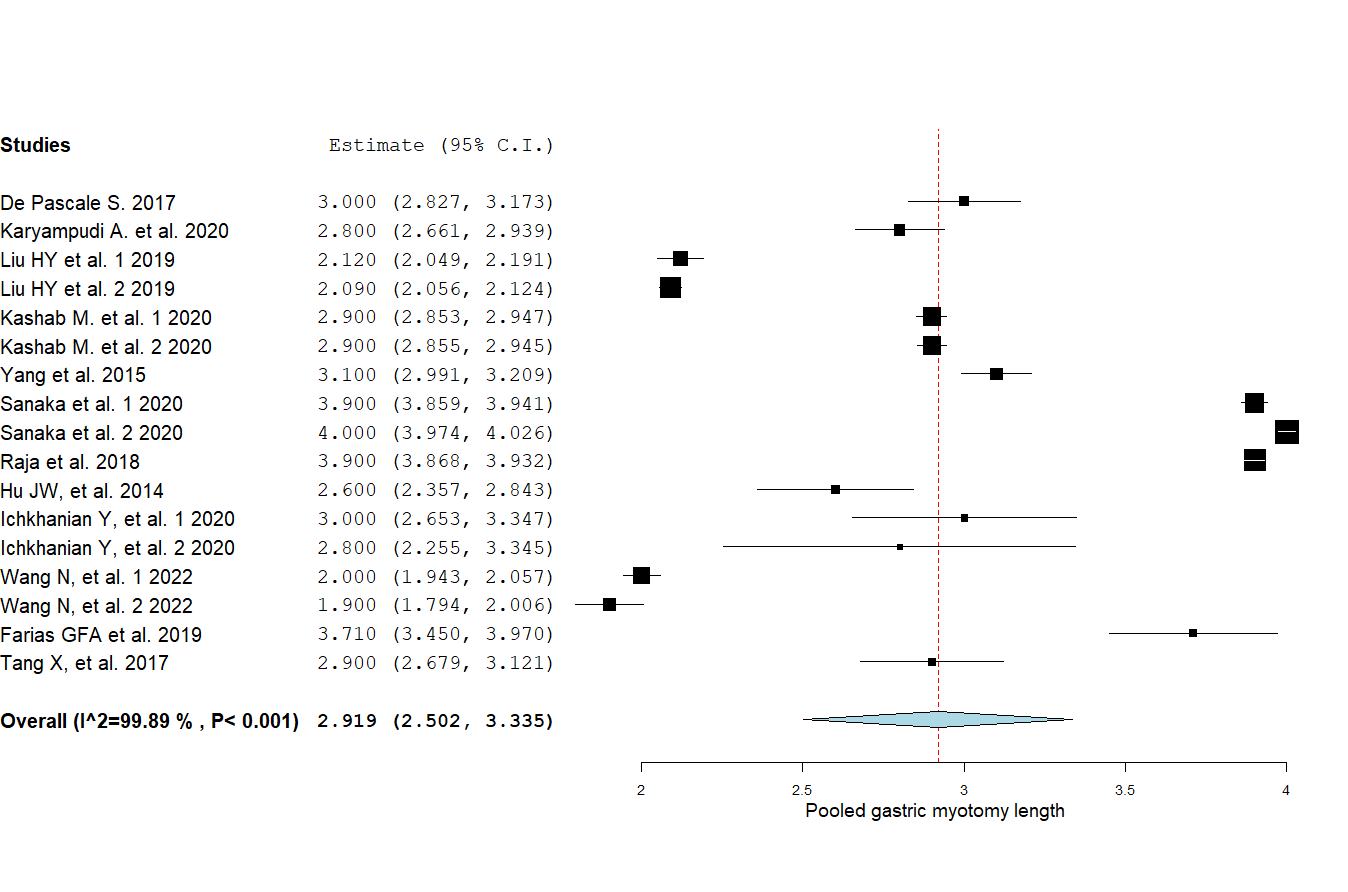

Supplement: Supplementary_data_doae069 [file supplementary_data_doae069.zip › Supplementary Figure 2B.png]

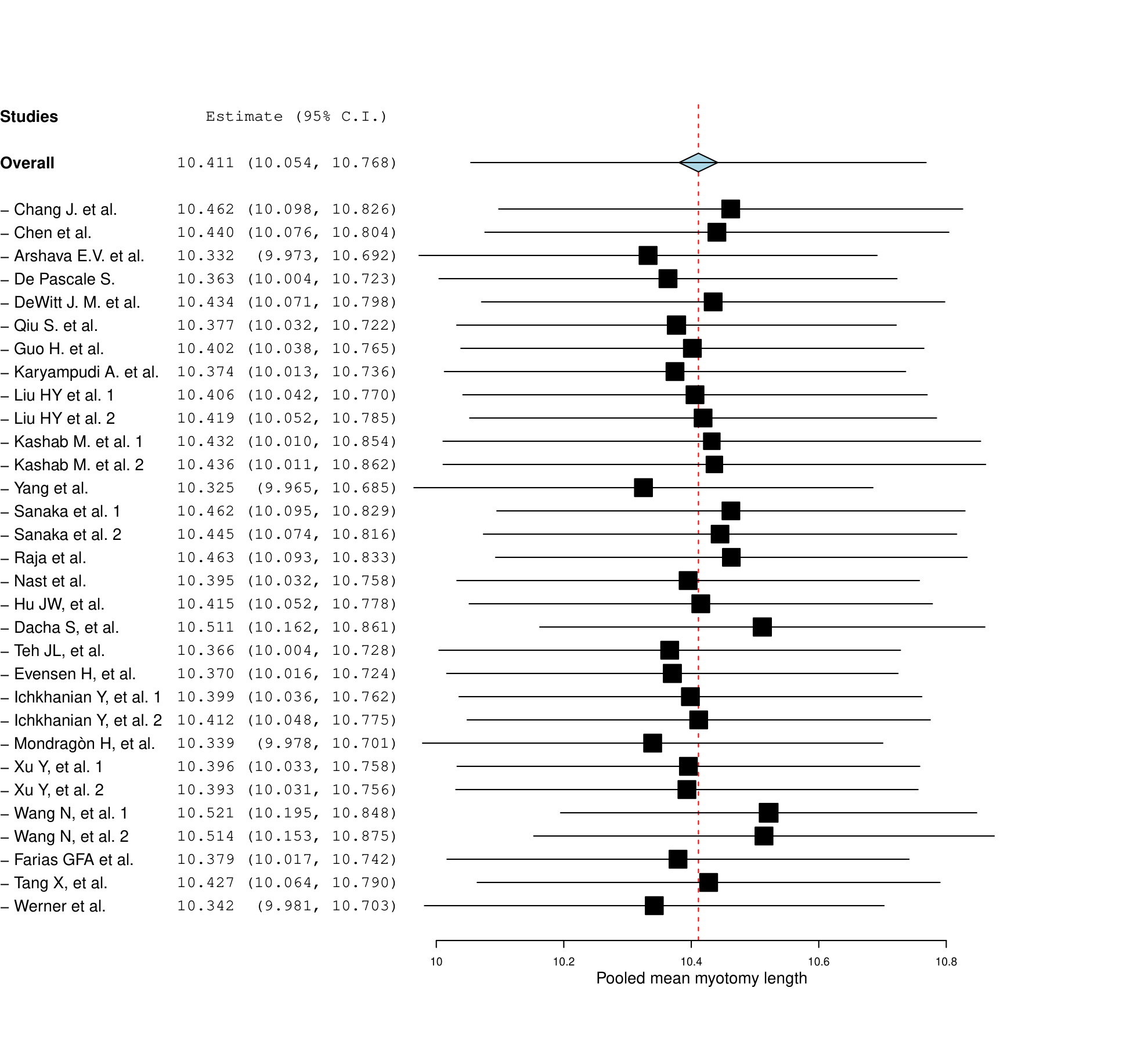

Supplement: Supplementary_data_doae069 [file supplementary_data_doae069.zip › Supplementary Figure 3.tiff]

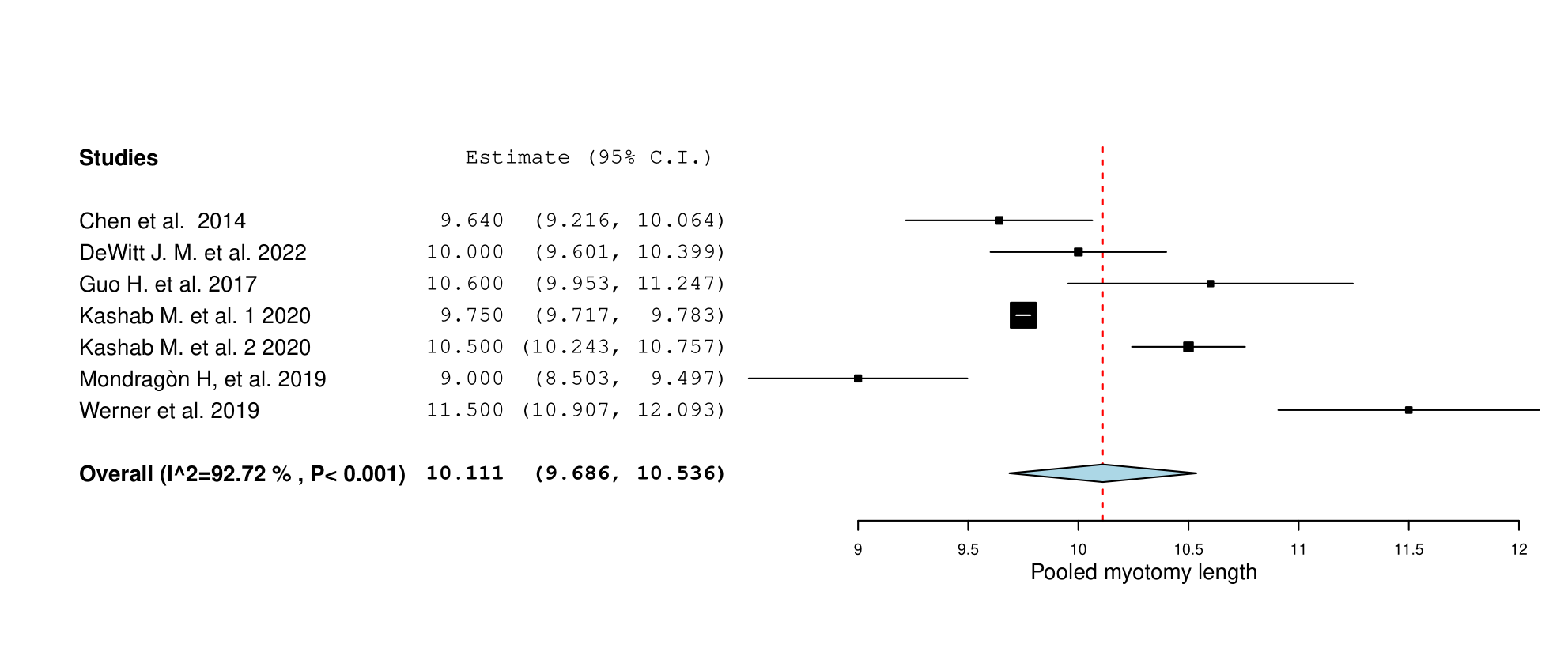

Supplement: Supplementary_data_doae069 [file supplementary_data_doae069.zip › Supplementary Figure 4A.png]

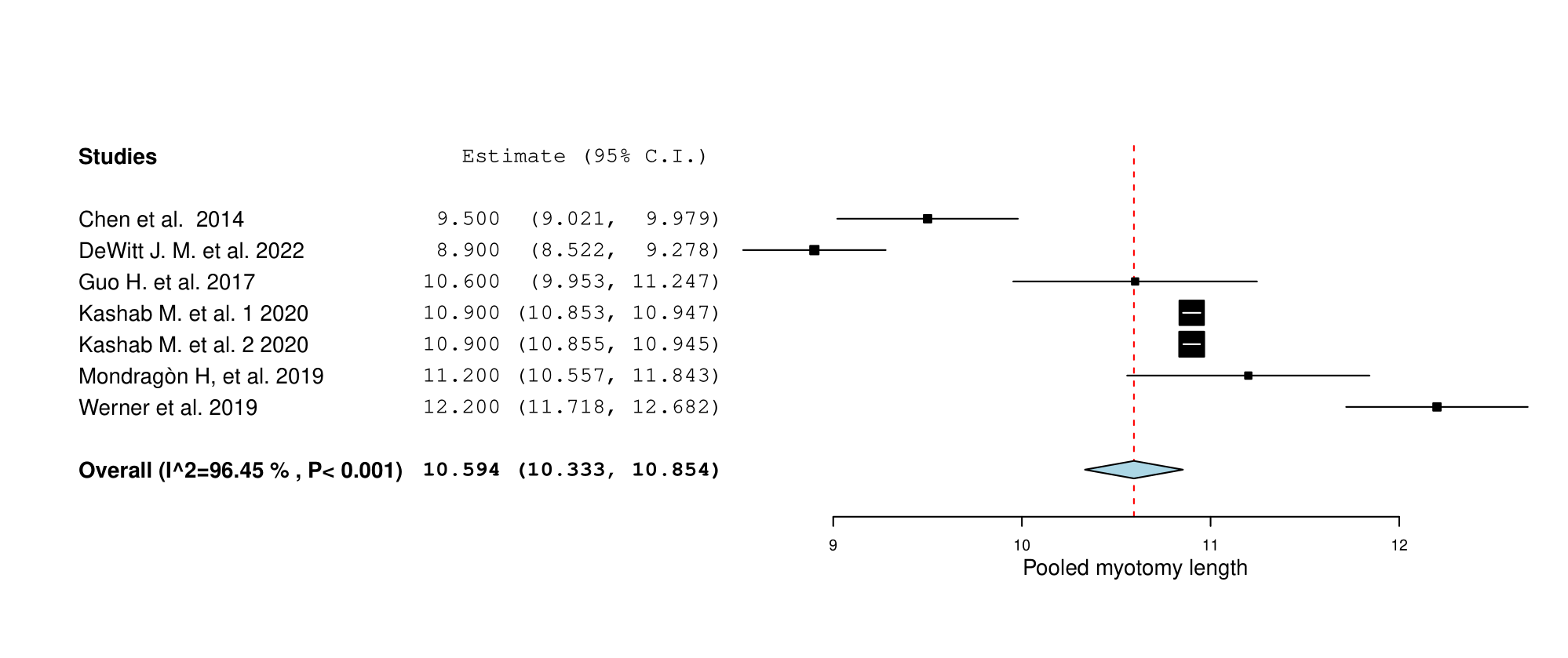

Supplement: Supplementary_data_doae069 [file supplementary_data_doae069.zip › Supplementary Figure 4B.png]

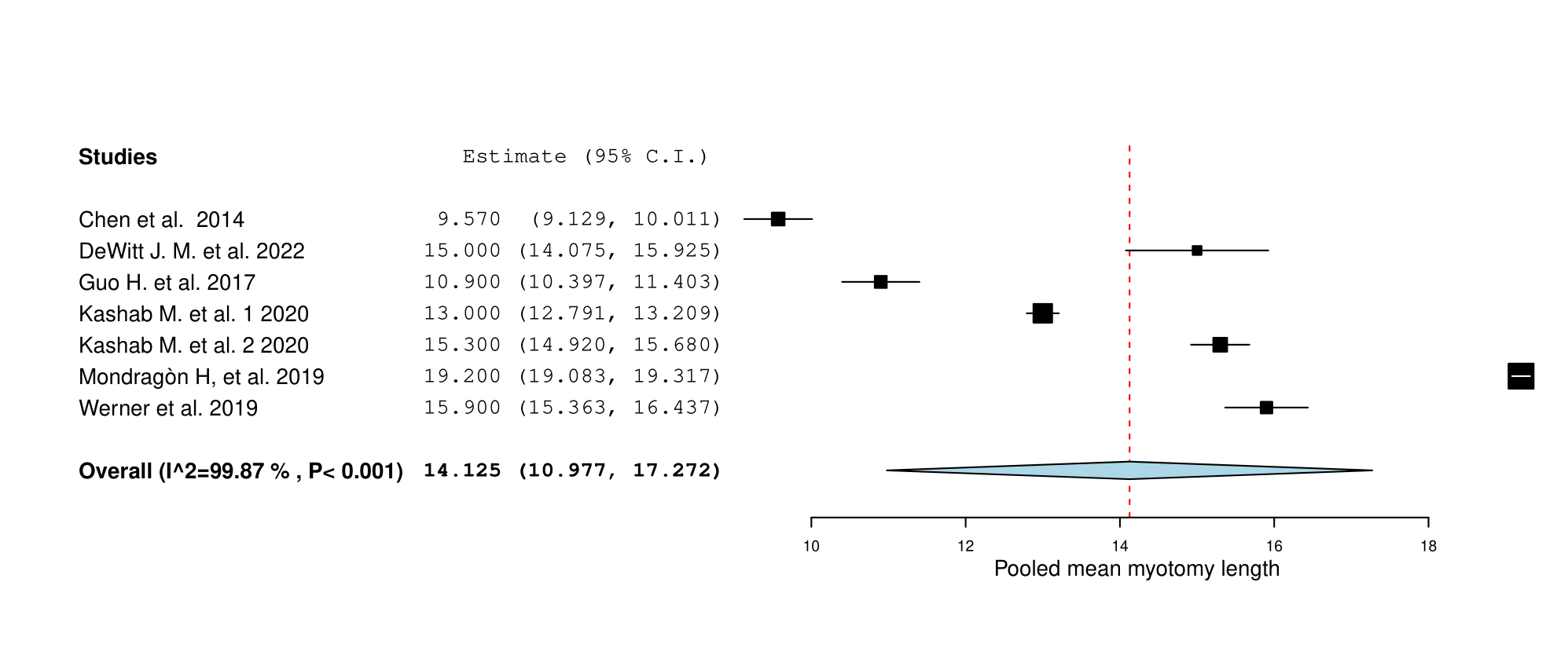

Supplement: Supplementary_data_doae069 [file supplementary_data_doae069.zip › Supplementary Figure 4C.png]

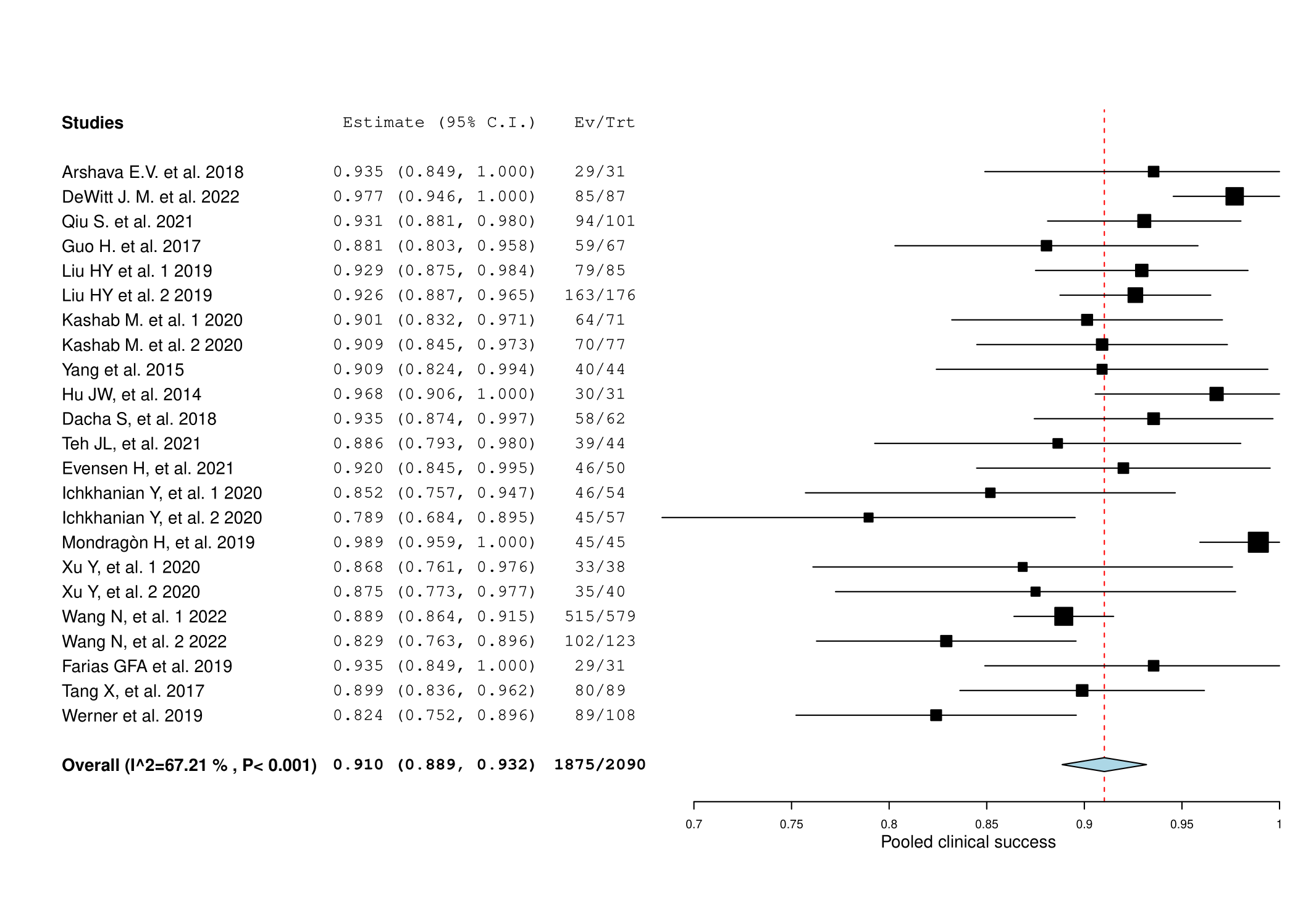

Supplement: Supplementary_data_doae069 [file supplementary_data_doae069.zip › Supplementary Figure 5.png]

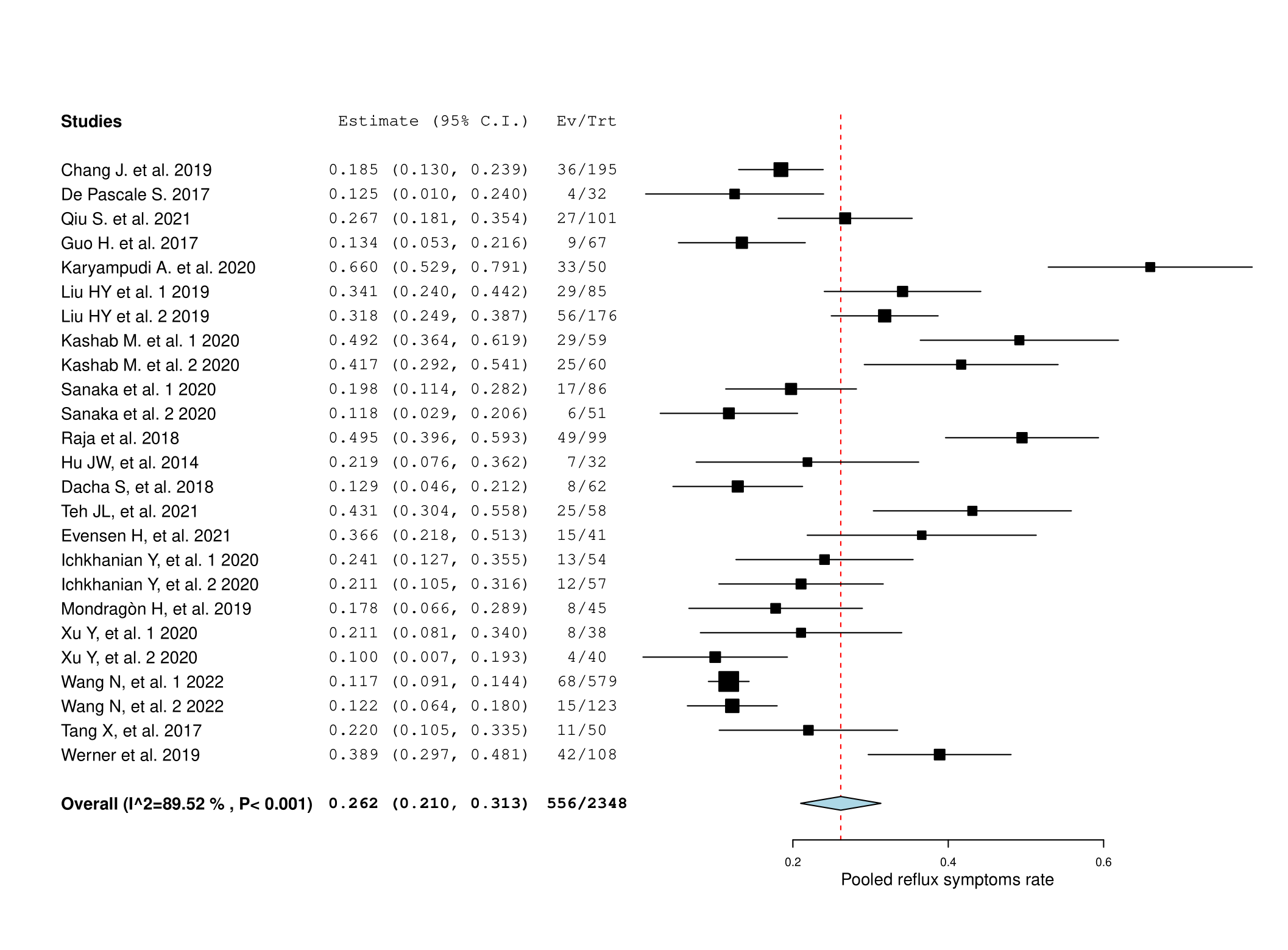

Supplement: Supplementary_data_doae069 [file supplementary_data_doae069.zip › Supplementary Figure 6.png]

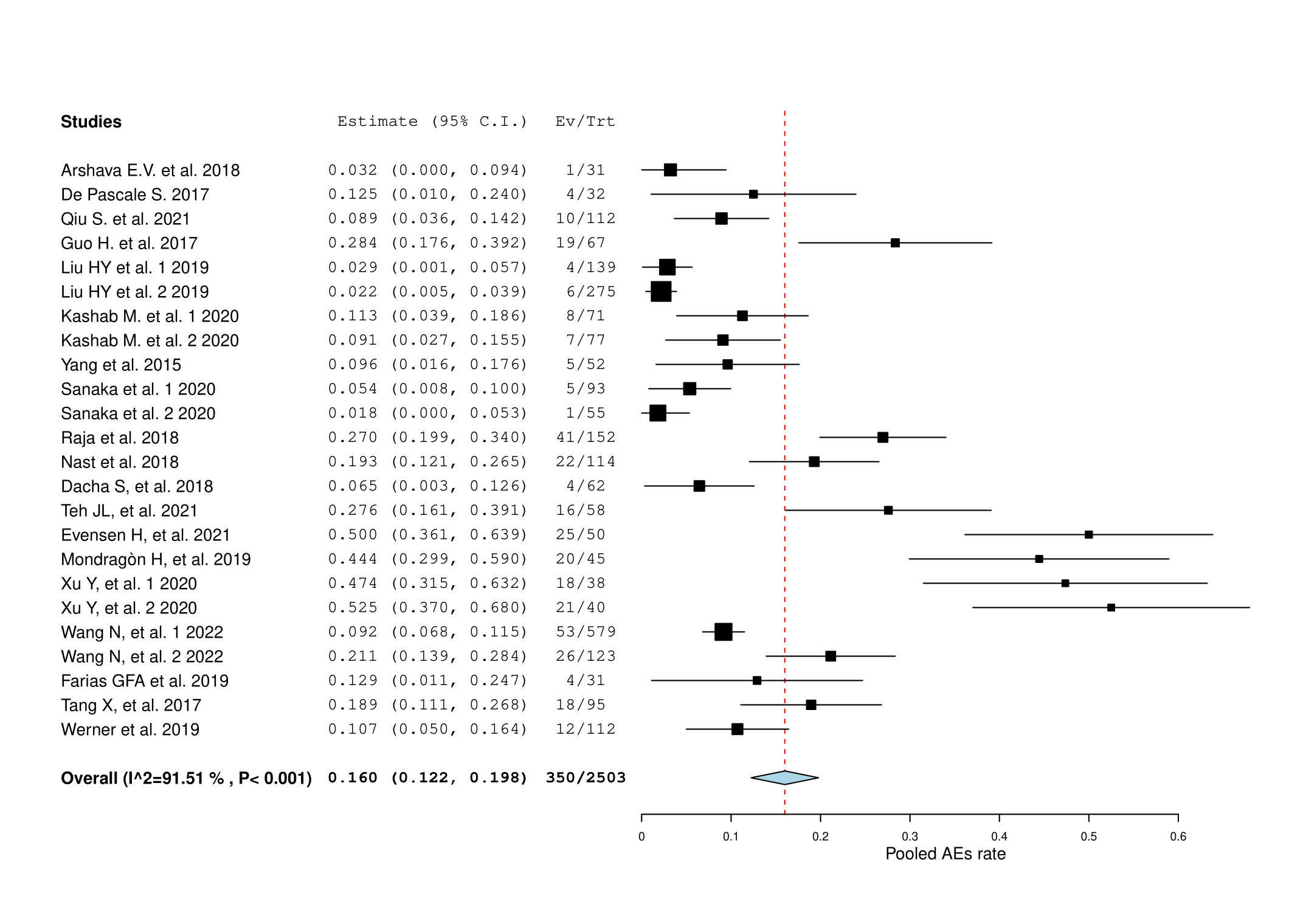

Supplement: Supplementary_data_doae069 [file supplementary_data_doae069.zip › Supplementary Figure 7A.png]

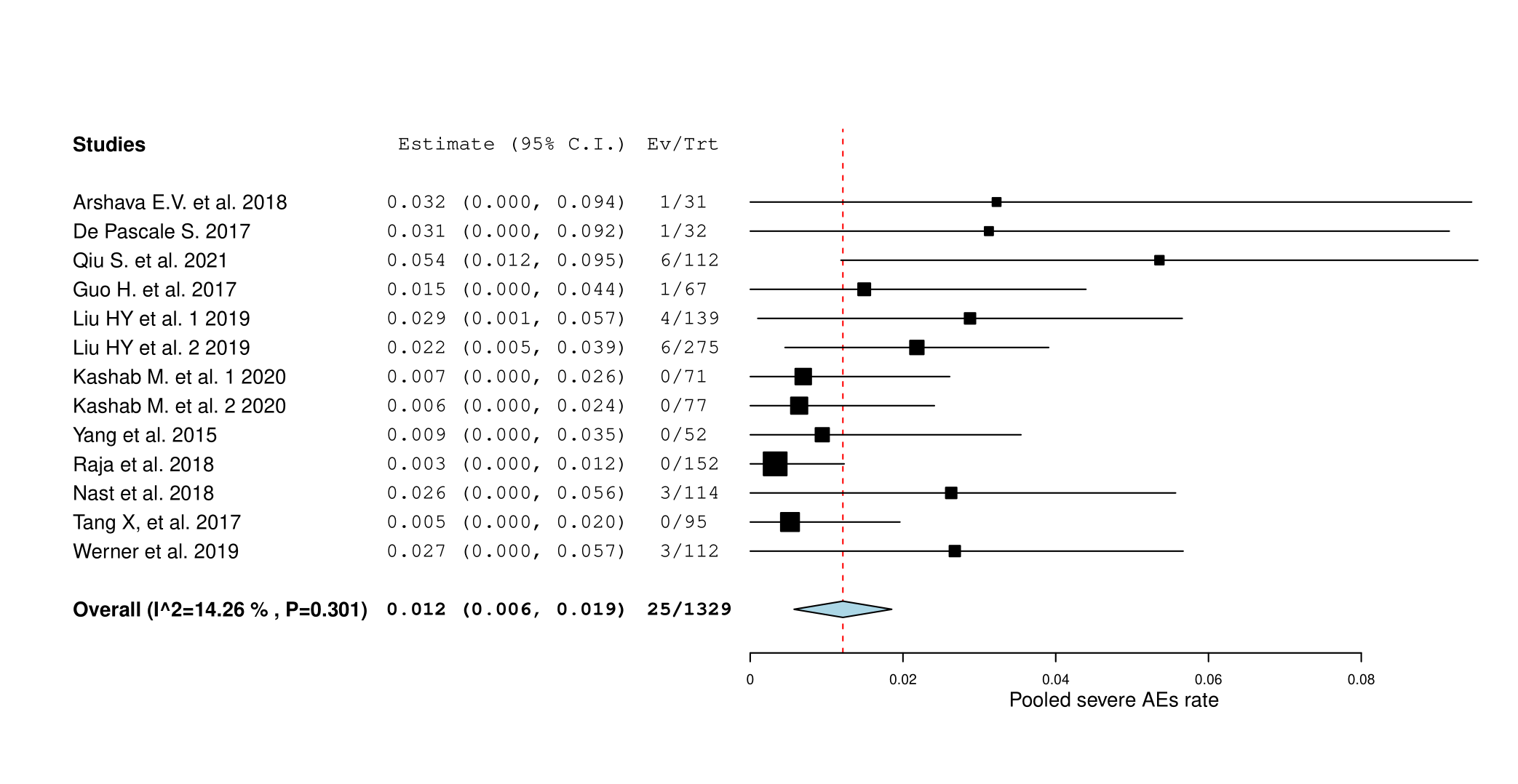

Supplement: Supplementary_data_doae069 [file supplementary_data_doae069.zip › Supplementary Figure 7B.png]

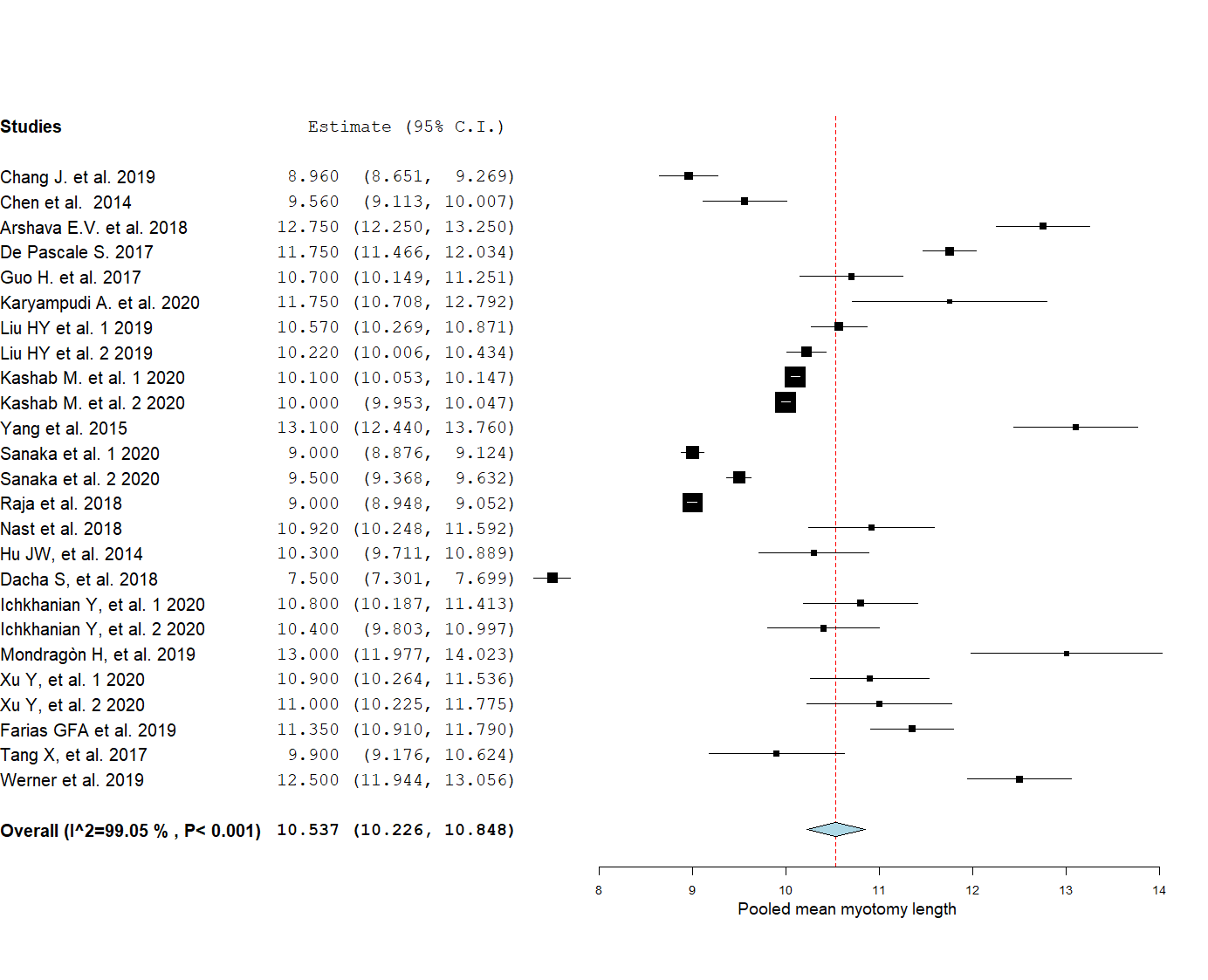

Supplement: Supplementary_data_doae069 [file supplementary_data_doae069.zip › Supplementary Figure 8A.png]

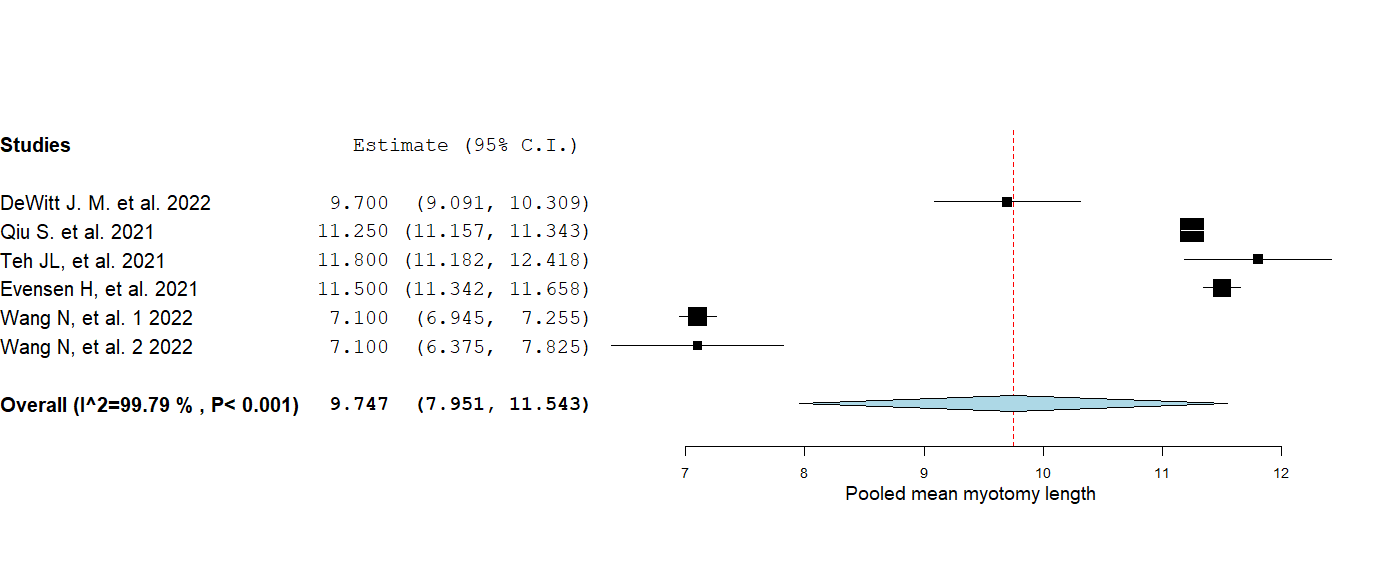

Supplement: Supplementary_data_doae069 [file supplementary_data_doae069.zip › Supplementary Figure 8B.png]
